# Changes in Transcriptome of *Yersinia pseudotuberculosis* IP32953 Grown at 3 and 28°C Detected by RNA Sequencing Shed Light on Cold Adaptation

**DOI:** 10.3389/fcimb.2018.00416

**Published:** 2018-11-27

**Authors:** Jussa-Pekka Virtanen, Riikka Keto-Timonen, Kaisa Jaakkola, Noora Salin, Hannu Korkeala

**Affiliations:** Department of Food Hygiene and Environmental Health, Faculty of Veterinary Medicine, University of Helsinki, Helsinki, Finland

**Keywords:** cold stress, stress tolerance, compatible solute, RNA helicase, cold shock protein, transcription factor

## Abstract

*Yersinia pseudotuberculosis* is a bacterium that not only survives, but also thrives, proliferates, and remains infective at cold-storage temperatures, making it an adept foodborne pathogen. We analyzed the differences in gene expression between *Y. pseudotuberculosis* IP32953 grown at 3 and 28°C to investigate which genes were significantly more expressed at low temperature at different phases of growth. We isolated and sequenced the RNA from six distinct corresponding growth points at both temperatures to also outline the expression patterns of the differentially expressed genes. Genes involved in motility, chemotaxis, phosphotransferase systems (PTS), and ATP-binding cassette (ABC) transporters of different nutrients such as fructose and mannose showed higher levels of transcripts at 3°C. At the beginning of growth, especially genes involved in securing nutrients, glycolysis, transcription, and translation were upregulated at 3°C. To thrive as well as it does at low temperature, *Y. pseudotuberculosis* seems to require certain cold shock proteins, especially those encoded by *yptb3585, yptb3586, yptb2414, yptb2950*, and *yptb1423*, and transcription factors, like Rho, IF-1, and RbfA, to maintain its protein synthesis. We also found that genes encoding RNA-helicases CsdA (*yptb0468*), RhlE (*yptb1214*), and DbpA (*yptb1652*), which unwind frozen secondary structures of nucleic acids with cold shock proteins, were significantly more expressed at 3°C, indicating that these RNA-helicases are important or even necessary during cold. Genes involved in excreting poisonous spermidine and acquiring compatible solute glycine betaine, by either uptake or biosynthesis, showed higher levels of transcripts at low temperatures. This is the first finding of a strong connection between the aforementioned genes and the cold adaptation of *Y. pseudotuberculosis*. Understanding the mechanisms behind the cold adaptation of *Y. pseudotuberculosis* is crucial for controlling its growth during cold storage of food, and will also shed light on microbial cold adaptation in general.

## Introduction

*Yersinia pseudotuberculosis* is an enteropathogenic bacterium that causes the foodborne infection yersiniosis. Although it has an optimum growth temperature of around 28°C, *Y. pseudotuberculosis* can grow and multiply at cold-storage temperatures, even as low as 0°C (Keto-Timonen et al., 2018). Long storage at low temperature favors the growth of psychrotrophs like *Y. pseudotuberculosis*, as they can proliferate without much competition. Outbreaks of *Y. pseudotuberculosis* have been associated with vegetables, raw milk, and drinking untreated water (Sato and Komazawa, 1991; Nuorti et al., 2004; Rimhanen-Finne et al., 2009; Pärn et al., 2015). In order to survive the various ecological niches through which it could enter the food chain, *Y. pseudotuberculosis* carries many tools in its genome, such as operons for the transport of substrates more abundant in plants and soil than animal tissue, as well as type VI secretion systems (Jaakkola et al., 2015). The bacterium has been found in the intestines of many animals like domestic pigs, goats, sheep, wild lagomorphs, birds, rodents, and shrews (Niskanen et al., 2003; Laukkanen et al., 2008; Giannitti et al., 2014; Le Guern et al., 2016; Joutsen et al., 2017). It can also thrive in soil as well as in certain protozoans and nematodes (Buzoleva and Somov, 2003; Gengler et al., 2015; Santos-Montañez et al., 2015).

Adaptation to cold and long-term growth at low temperatures poses many challenges to bacteria. Low temperature decreases the fluidity of cell membranes, thereby interfering with normal membrane protein function. It also slows down protein folding, ribosomes, and translation as well as excessively stabilizes nucleic acid structures (Palonen et al., 2010). Cold also induces radical oxygen species production by both slowing metabolism and increasing oxygen solubility (Chattopadhyay et al., 2011). Psychrotrophic bacteria have many methods to deal with these problems. For example, bacteria can increase membrane fluidity by introducing unsaturated lipids that no longer fit as snugly together (Suutari and Laakso, 1994). Unsaturated fatty acids dominate the fatty acid composition of *Y. pseudotuberculosis* at low temperatures (Bakholdina et al., 2004).

Cold shock proteins (Csp) are small proteins whose mRNA carries a cold shock domain that enables its translation at low temperatures. Csps, along with helicases and translation factors exhibiting similar stabilizing secondary structures, unwind mRNA and support the translational apparatus at low temperatures. *Y. pseudotuberculosis* has nine *csp* genes, homologous to those of *Escherichia coli*, five of which are induced at low temperature in *E. coli* (Keto-Timonen et al., 2016). The bacterium also has five helicases with a conserved DEAD-box motif: CsdA (*yptb0486*), RhlE (*yptb1214*), RhlB (*yptb0165*), DbpA (*yptb1652*), and SrmA (*yptb2900*).

The main goal of this study was to determine how *Y. pseudotuberculosis* manages to thrive at refrigerator temperature. We identified which genes showed significantly more transcripts at 3°C when compared to 28°C, at each growth phase, mainly focusing on the beginning of growth and logarithmic phase. Of all cold shock proteins, those encoded by *yptb1423, yptb3585, yptb3586*, and especially *yptb2414* and *yptb2950*, showed significantly more transcripts at low temperature, seemingly forming the backbone of cold acclimation of *Y. pseudotuberculosis*. Furthermore, we found that, in addition to CsdA, helicases RhlE, and DbpA were significantly upregulated at low temperature, which speaks to their importance in surviving low temperatures. The bacterium also seems to accumulate glycine betaine by uptake and biosynthesis, as the corresponding genes were upregulated at low temperature. Transcription termination factor Rho, along with IF-1 and RbfA, both acting on ribosomes, were also upregulated, which would seem to suggest that they play an important role. None of these genes and proteins have, to our knowledge, been linked to cold acclimation of *Y. pseudotuberculosis* before.

## Materials and methods

### Bacterial strain and growth conditions

**Figure 1:**
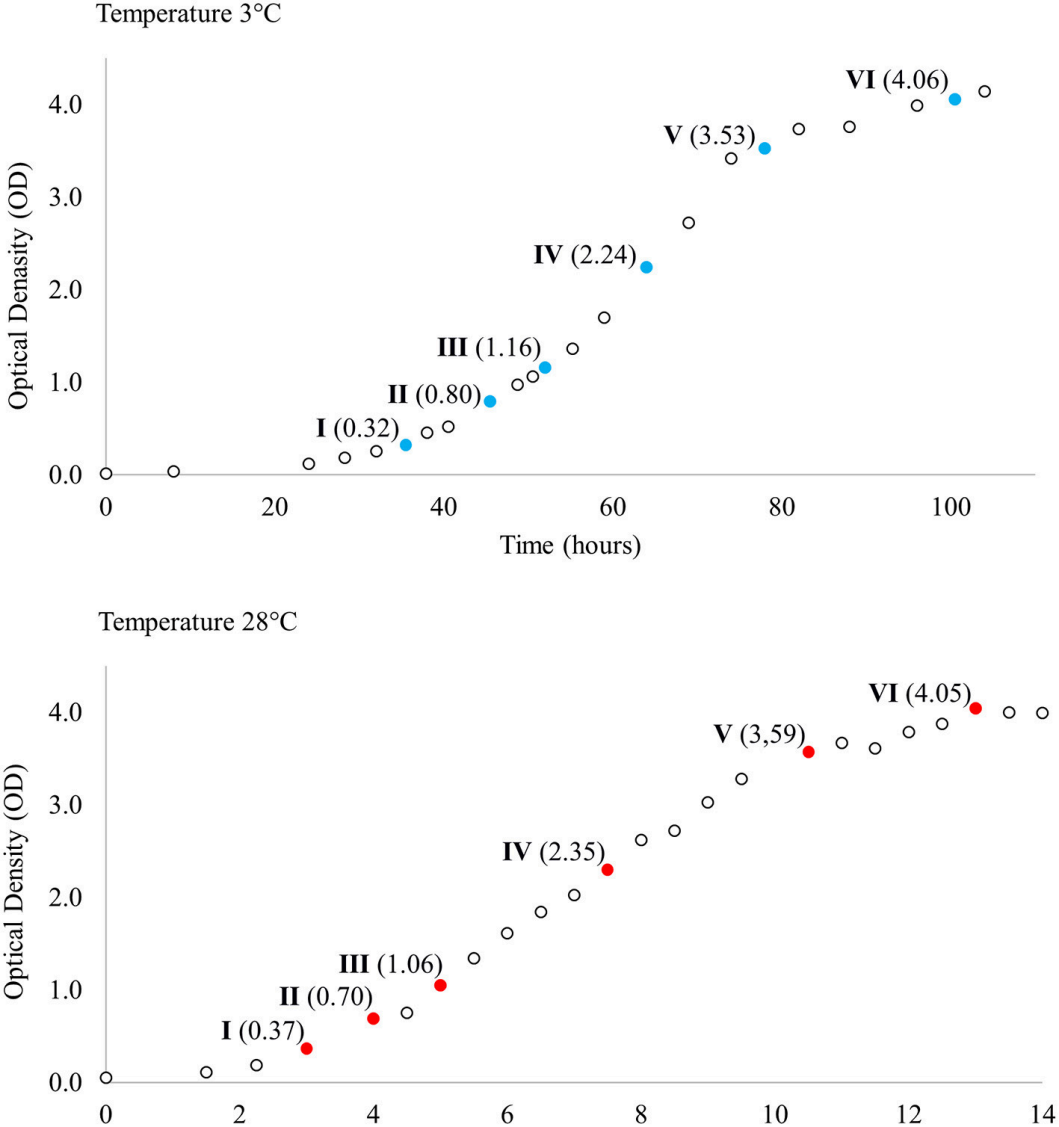
Growth curves of *Yersinia pseudotuberculosis* IP32953 grown at 3 and 28°C. Samples for total RNA extraction and sequencing were collected at both temperatures at six corresponding growth points I–VI selected by optical density (OD).

Single *Y. pseudotuberculosis* IP32953 colonies grown on blood agar plates at 28°C were inoculated and grown separately in LB broth (Luria-Bertani; Sigma-Aldrich, St. Louis, MO, USA) at 28°C with shaking overnight. Overnight broths were diluted (1:100) in LB broth and divided into two groups so that four cultures (biological replicates) were grown with shaking both at 3°C and at 28°C. Biological replicates were used to better model true biological variability and improve the accuracy of statistical methods. Samples for total RNA extraction were collected at six corresponding points at different phases of growth across both temperatures (Figure 1). For total RNA extraction 1.25 ml of bacterial culture was mixed with 250 μl cold phenol-ethanol mixture (1:10) and kept on ice for 30 min. After incubation, samples were centrifuged at 4°C at 13,200 rpm for 2 min and the resulting cell pellets were stored at −70°C until RNA isolation.

### RNA isolation

Total RNA was isolated using GeneJET RNA Purification Kit (Thermo Fisher Scientific, Waltham, MA, USA) and treated with DNA-Free DNA Removal Kit (Ambion, Life Technologies, Carlsbad, CA, USA) according to manufacturers' instructions. The quantity and quality of RNA was examined with a Nanodrop ND-1000 Spectrophotometer (Thermo Fisher Scientific) and Agilent 2100 Bioanalyzer (Agilent Technologies, Santa Clara, CA, USA). RNA was stored at −70°C until RNA-seq library preparation.

### Library preparation and sequencing

Ribosomal RNA was depleted from total RNA using Ribo-Zero rRNA Removal Kit for Bacteria (Epicenter, Madison, WI, USA) following the manufacturer's protocol. cDNA libraries were prepared using Script-Seq v2 RNA-Seq Library Preparation Kit (Epicenter) following the manufacturer's instructions. The libraries were amplified twice by PCR and barcoded. PCR was performed using Phusion High-Fidelity DNA Polymerase Kit (Thermo Fisher Scientific). The libraries were purified using AMPure XP System (Beckman Coulter, Brea CA, USA) after both PCR procedures. The libraries were sequenced (75 bp single read) using the Illumina NextSeq500 platform at the Institute of Biotechnology, University of Helsinki, yielding four sets of sequence reads (biological replicates) per growth point at both 3 and 28°C. Raw sequences were deposited in the Sequence Read Archive (http://www.ncbi.nlm.nih.gov/sra) under accession number SRP144570.

### CDNA synthesis and RT-qPCR

To confirm the differences of transcript levels identified in the RNA-sequence expression analysis, RT-qPCR validation was performed for selected genes (*betB, csdA, fabF, infA, mdtI, proX, rhlE*, and *rho*) at growth points I and III at both temperatures (3 and 28°C). These genes or operons they represent are discussed in detail in this paper. A total of 500 ng of each RNA sample from three biological replicates per growth point was reverse-transcribed into cDNA in duplicate by using Maxima cDNA Synthesis Kit (Thermo Fisher Scientific) according to manufacturer's instructions. Primers for RT-qPCR were designed using Primer-BLAST software ((Ye et al., 2012); Table 1). Two replicate qPCR reactions for each cDNA sample were performed using the Dynamo Flash SYBR Green qPCR Kit (Thermo Scientific). Each reaction consisted of 1x Master Mix, 0,5 μM of forward and reverse primer and 4 μl of 1:20 (gene of interest) or 1:100,000 (16S *rrn*) diluted cDNA in a total volume of 20 μl. Rotor-Gene Q thermal cycler (Qiagen GmbH, Hilden Germany) was used in PCR runs with the cycling protocol consisting of initial heating step at 95°C for 7 min, followed by 40 cycles of denaturation at 95°C for 10 s, annealing at 60°C for 15 s, extension at 72°C for 20 s, and a final extension at 60°C for 1 min. After each run a melt curve analysis was done to confirm specificity. Amplification reaction efficiencies for each primer pair were obtained from RT-qPCR standard curves prepared from serial dilutions of pooled cDNA samples. The duplicate C_q_ values for PCR replicates were averaged. The relative quantification of gene of interest transcript levels at 3°C, normalized to reference gene (16S *rrn*) transcript levels and calibrated to the samples taken at the same growth point at 28°C, were calculated using the Pfaffl method (Pfaffl, 2001). Given near equal primer efficiencies, the logFC values of Pfaffl gene expression ratios between 3 and 28°C should be proportional to the logFC values acquired by RNA-Seq. A linear regression analysis was performed on corresponding logFC values of RNA-Seq and RT-qPCR.

**Table 1:** Primers used in quantitative real-time reverse transcription-PCR.

**Gene**	**Forward primer (5^′^ → 3^′^)**	**Reverse primer (5^′^ → 3^′^)**
16S rRNA gene	GCTCGTGTTGTGAAATGTTGG	TATGTGGTCCGCTGGCTCT
*betB*	AGGATTTGAACCGTGCCCAT	GCTGATACCGTTTTCACGGC
*csdA*	GTGATGTTGGCGAGATGGAG	ATCGTTGAGTGGGAAGCAAA
*fabF*	ATGCTGATGTCATGGTGGCT	TGCTGCTTGTGGGTTATCGT
*infA*	TCTTGATACGCTGCCGAACA	TGACTTTGTCACCCGTCAGG
*mdtI*	CCCGCCAGCAGTAAGATCAA	ATGTGGGGCGGTTTTGGTAT
*proX*	GATAACCCTGTGCCAAGCCT	GATGCAACCTATCTGGCGGT
*rhlE*	ATAGCGGCAAGGTGAAACCA	ATCCTGCGTGCTGTTGAAGA
*rho*	CGTTAGTTTTCGCAGTCGCC	CCGCTCTGGTACCCGTAAAG

### Alignment and annotation of RNA-seq data

We aligned, annotated, and analyzed the sequence reads with Bioconductor (Huber et al., 2015). The complete reference genome sequence and genomic features of *Y. pseudotuberculosis* IP32953 (Johnson et al., 2015) were acquired from the Pathosystems Resource Integration Center, PATRIC (Wattam et al., 2014). The sequence reads were both aligned to the reference genome, allowing a maximum of 10 hits, and annotated with Bioconductor package QuasR (Gaidatzis et al., 2015).

### Differential gene expression analysis

One of the replicates of growth point IV at 28°C was discarded for its low alignment quality. The remaining annotated transcript counts were analyzed using Bioconductor package baySeq (Hardcastle and Kelly, 2010). Low count reads were filtered out. The replicate counts for each growth point at 3°C were compared to the counts of the respective growth point at 28°C. The prior distributions were acquired using a negative binomial distribution whose parameters were estimated by quasi-maximum-likelihood methods with a sample size of 10,000. The posterior likelihoods were established using 10 iterations to re-estimate the priors. Results were normalized by library size and results with FDR >0.05 were discarded. Log_2_ fold changes (logFC) across the two temperatures were calculated using transcript count averages of replicates, and genes with logFC ≥ 2 were considered significantly expressed at 3°C. For ease of viewing, genes were further grouped into operons retrieved from ProOpDB (Taboada et al., 2012). Amino acid sequence similarities were derived from multiple sequence alignment by Clustal Omega (Sievers et al., 2011). Upregulated genes sharing similar functions were classified into functional units (modules), acquired from the KEGG database (Kanehisa and Goto, 2000), using Bioconductor package clusterProfiler (Yu et al., 2012). *P*-values were adjusted for multiple comparisons by controlling the false discovery rate, using the Benjamini & Hochberg method implemented in clusterProfiler (Benjamini and Hochberg, 1995).

### Clustering

**Figure 2:**
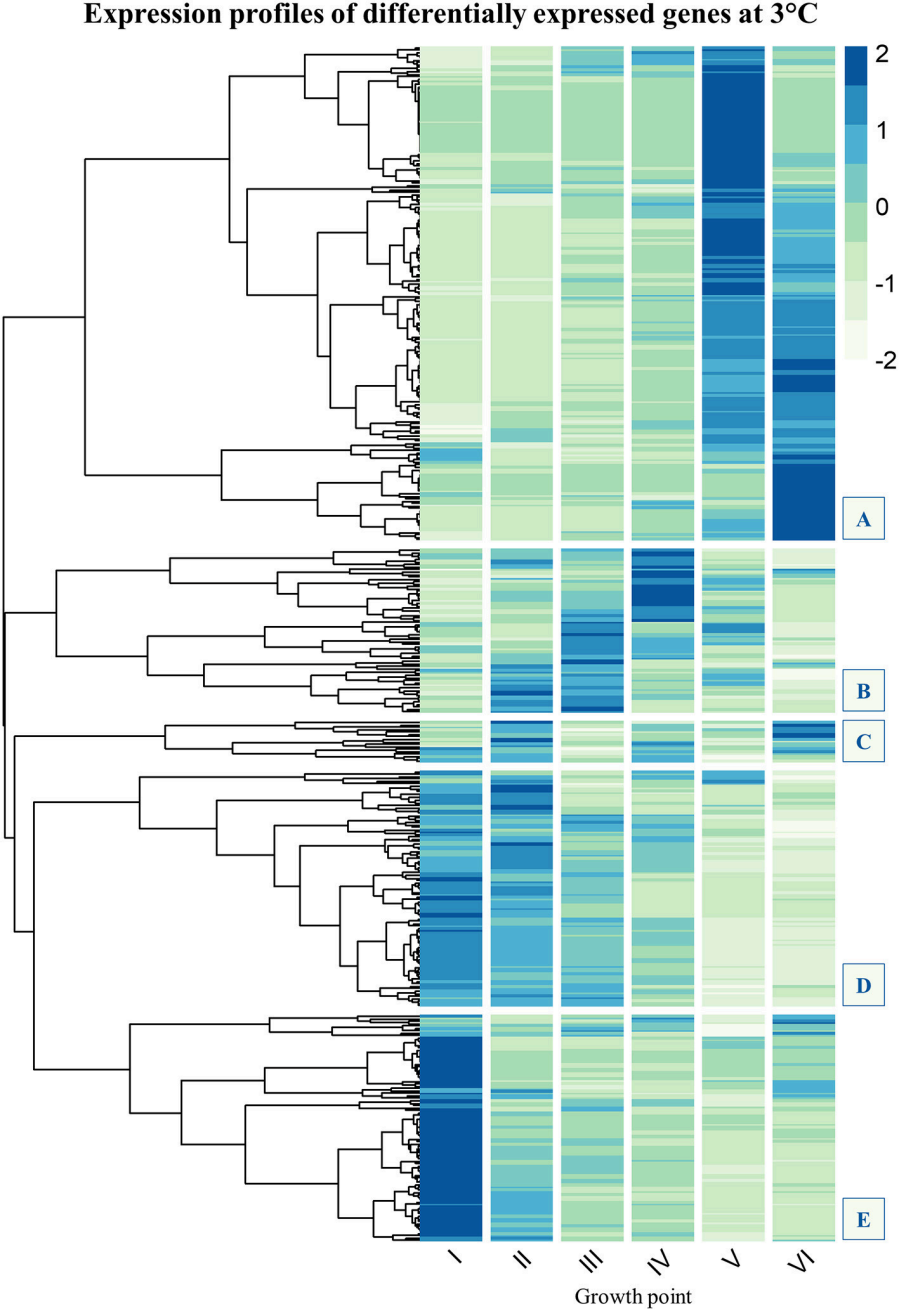
Gene expression profile clustering of *Yersinia pseudotuberculosis* strain IP32953 genes that showed significantly more transcripts (logFC ≥ 2, FDR ≤ 0.05) at 3°C than 28°C at least once during growth. The tree has been cut to five subclusters **(A–E)**. Subclusters **(B–E)** are of most interest because they comprise genes that express highly at the beginning and logarithmic phase of growth. Subcluster A consists of genes whose expression mainly peaks at stationary phase. Flagellar assembly (Figure S1) and chemotaxis genes (Figure S2) have been left out as *Y. pseudotuberculosis* is not motile at 28°C. In addition, tRNA-genes (Figure S3) have been filtered out, so that the final *N* = 482. The darker the color in the heatmap, the more transcripts the gene showed at that growth point.

Expression profiles of genes that were significantly more expressed at low temperature, at least at one growth point, were clustered using Pearson correlation distance. Count data was normalized for visualization by using median ratio normalization implemented in Bioconductor package DESeq2 (Love et al., 2014). Averages of replicate counts were used instead of individual counts. Normalized data for each gene (rows) across all growth points (columns) was then plotted in a heatmap (Figure 2).

## Results

Expression profiles of *Y. pseudotuberculosis* IP32953 grown at 3 and 28°C were compared at corresponding phases of the growth based on growth curves determined by the optical density. We found 570 genes in total that showed significantly more transcripts at 3°C than 28°C at least at one of the growth points I–VI (Table 2). Motility and chemotaxis genes were at the top throughout growth since *Y. pseudotuberculosis* is non-motile at 28°C. The total number of upregulated genes was 482 when motility, chemotaxis, and tRNA genes were filtered out. Growth point VI held the fewest significantly expressed genes (*N* = 125) whereas growth points II and IV held the most (*N* = 162; Table 2). The difference in expression was largest for genes involved in motility, with a maximum logFC of 7.42.

**Table 2:** The number of *Yersinia pseudotuberculosis* strain IP32953 genes expressed significantly more at 3°C than at 28°C by growth point.

**Growth point (OD)**	**logFC**	
	**≥2**	**≥4**
**I** (0.32–0.37)	134	27
**II** (0.69–0.80)	162	13
**III** (1.05–1.17)	139	9
**IV** (2.24–2.30)	162	9
**V** (3.53–3.57)	129	7
**VI** (4.05–4.06)	125	8

*Flagellar assembly and chemotaxis genes have been disregarded as Y. pseudotuberculosis is not motile at 28°C; and tRNA-genes have also been filtered out*.

A gene that was expressed both differentially and in great numbers at 3°C probably plays an important role in cold growth. We clustered expression profiles of genes that at least once during growth showed more transcripts at 3°C than at 28°C. Five subclusters (A–E) could be identified in the expression profile heatmap (Figure 2). The genes of most interest in the context of cold growth express highly at the beginning and logarithmic phases of growth, clustered into subclusters B–E (see details in Supplementary Material). Subcluster A mostly held genes that were expressed highly at the stationary phase.

### Validation of RNA sequencing results with RT-qPCR

**Figure 3:**
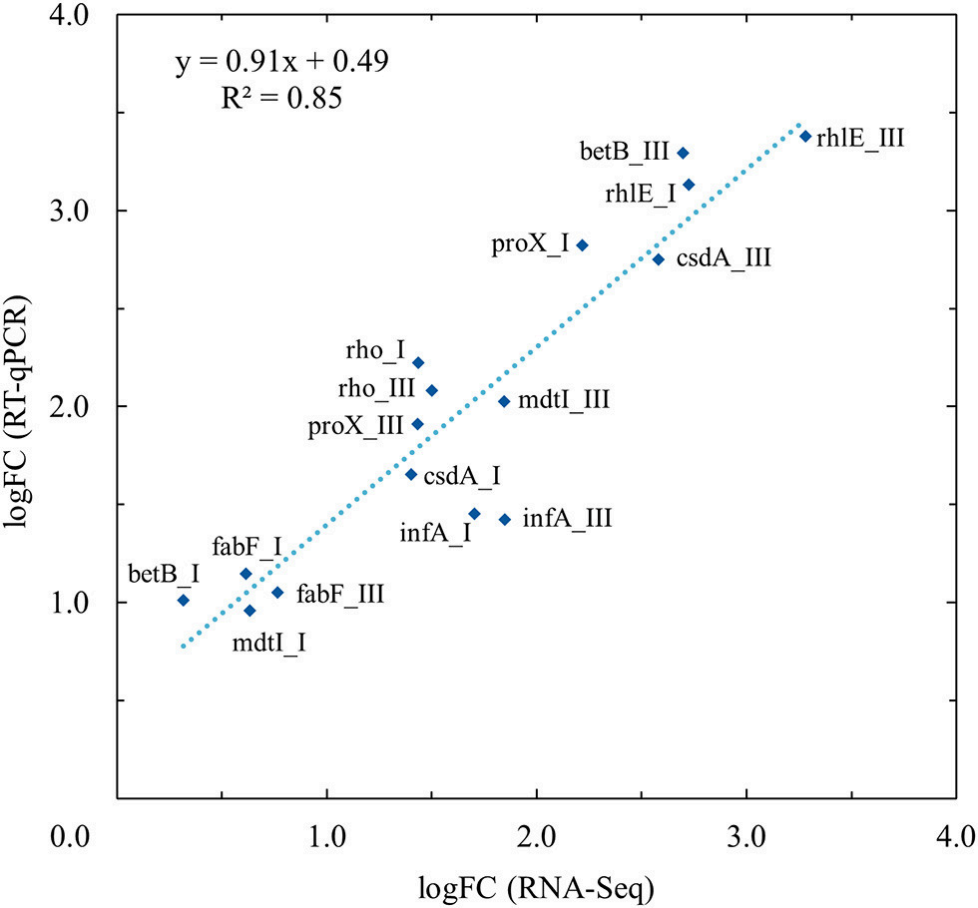
Validation of RNA-Seq results with quantitative real-time reverse-transcription PCR (RT-qPCR) using linear regression analysis. RT-qPCR validation was performed for selected genes (*betB, csdA, fabF, infA, mdtI, proX, rhlE*, and *rho*) at growth points I and III at both temperatures (3 and 28°C). Linear regression analysis showed an *R*^2^ coefficient of determination value of 0.85 between the RNA-Seq and RT-qPCR log_2_ fold changes (logFC).

The expression profiles of eight differentially expressed genes at two different growth points were evaluated by RT-qPCR and compared with the RNA-seq analysis results. In linear regression analysis between the RNA-seq and RT-qPCR logFC values (Figure 3), a 0.93 Pearson correlation coefficient value (*R*^2^ = 0.85) was observed, which confirmed the reproducibility and reliability of the RNA-seq method (Figure 3).

### Differentially expressed genes by growth point

**Figure 4:**
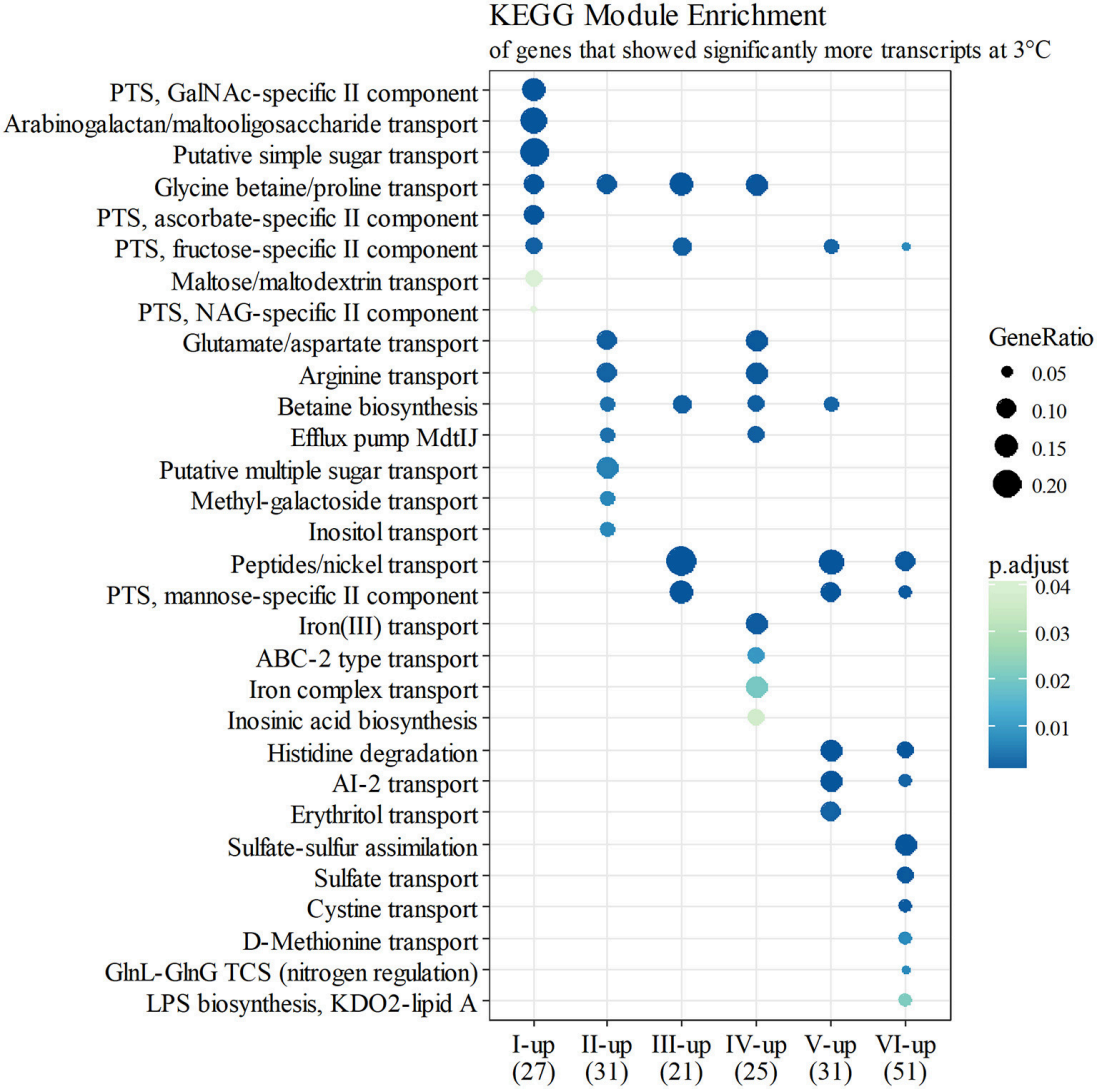
KEGG module enrichment (BH adjusted *p* ≤ 0.05) of genes that showed significantly more transcripts at 3°C (logFC ≥ 2 for at least one operon member, FDR ≤ 0.05). A gene can belong to multiple modules, or none. Some module names have been shortened for clarity.

**Figure 5:**
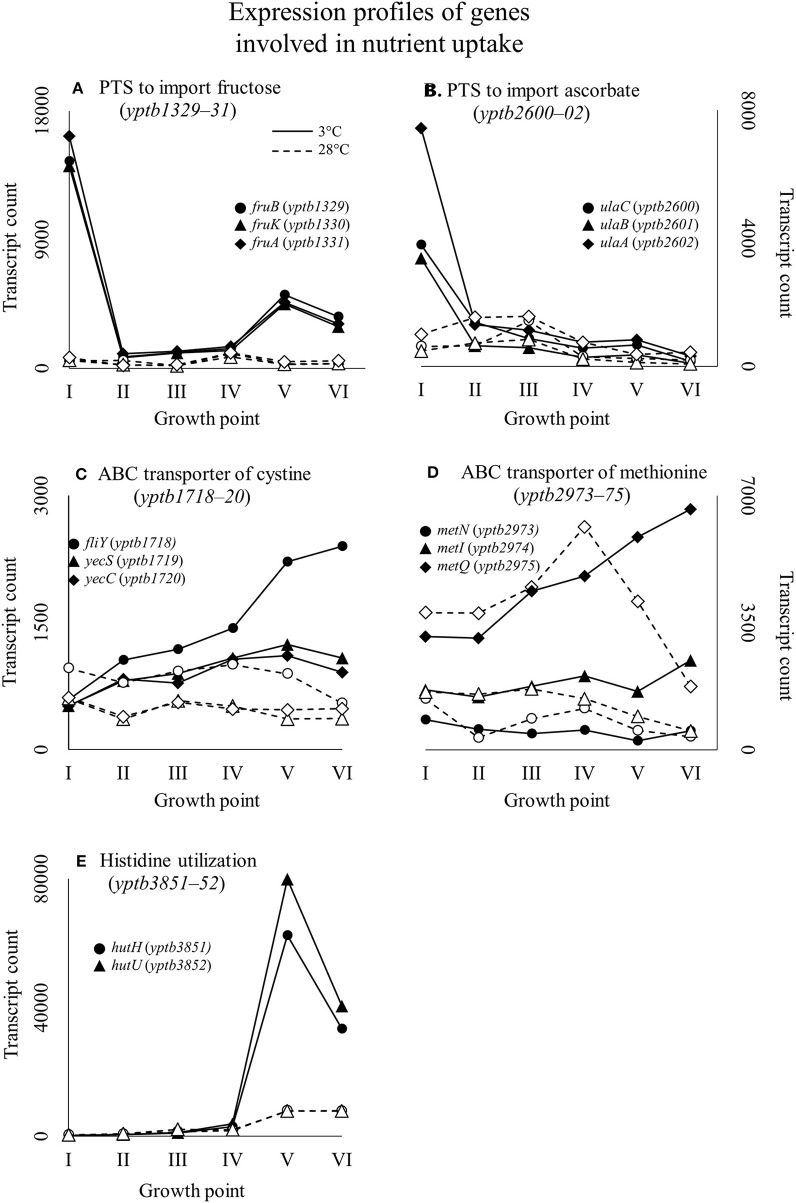
Expression profiles of example genes involved in nutrient uptake: *fru*-operon encoding a phosphotransferase system (PTS) to import fructose **(A)***; ula*-operon encoding a PTS to import ascorbate **(B)**; ATP binding cassette (ABC) transporter of cystine, dimer form of cysteine **(C)**; ABC transporter of methionine **(D)**; and part of the histidine utilization system **(E)** of *Yersinia pseudotuberculosis* IP32953, that was grown at 3 and 28°C and sampled at corresponding growth points I–VI. Median ratio normalized counts were used. A large significant difference in expression of sugar importers between 3 and 28°C can be observed at the beginning of growth. Toward the end of growth at 3°C, amino acid utilization takes precedence when sugar importer expression tapers off.

At growth point I, genes involved in acquiring compatible solutes and various nutrients showed significantly more transcripts at 3°C, and this pattern continued until stationary phase (Figure 4). For example, a significant increase in transcripts at 3°C was displayed in the following genes: genes encoding a glycine betaine transporter (*yptb2959–61*); phosphotransferase systems (PTS) to import fructose (*yptb1329–31*; Figure 5), N-acetylglucosamine (*yptb1120, yptb3075–82*), and L-ascorbate (*yptb2600–2*; Figure 5); and ATP-binding cassette (ABC) transporters of maltose (*yptb2521, yptb3095–102*) and aldopentoses (*yptb3591–6*). A significant portion of upregulated genes at this growth point were associated with different PTSs and glycine betaine transport (Figure 4). As expected, genes encoding chaperone molecules such as helicase RhlE (*yptb1214*) and Csps (*yptb2950, yptb2414, yptb3585*, and *yptb3586*), which destabilize nucleic acid secondary structures, showed more transcripts at 3°C.

At growth point II, genes involved in further processing of compatible solutes, spermidine efflux, synthesizing desaturated membrane lipids, biosynthesis of ribosomes, securing translation under cold stress, and posttranscriptional modification of RNA molecules showed significantly more transcripts at 3°C. Modules of different amino acid transporters and betaine synthesis were overexpressed (Figure 4). Genes encoding translation factors IF-1 (*yptb1395*) and Rho factor (*yptb0167*) peaked at this growth point. New additions to differentially expressed chaperone genes were helicase gene *dbpA* (*yptb1652*) and a new Csp gene (yptb1423).

At growth point III, a urease operon (*yptb2938–44*) and PTSs to import fructose (Figure 5), N-acetylglucosamine, and mannose showed significantly more transcripts at 3°C than 28°C. The gene encoding superoxide dismutase (*yptb3925*) also showed more transcripts at 3°C at this growth point. At growth point IV, it seems the nutrient strategy shifted as an operon involved in sulfur metabolism (*yptb2309–14*) was expressed more at 3°C than 28°C. In addition to genes encoding IF-1 and Rho factor, a new translation factor gene *rbfA* (*yptb0481*) showed more transcripts at 3°C at this point of growth.

At growth points V and VI, the nutrient strategy continued shifting as operons involved in the metabolism of histidine (*yptb1965–9, yptb3851–2*; Figure 5), cystine (*yptb1717–20*; Figure 5), methionine (*yptb2973–5*; Figure 5), and nitrogen compounds (*yptb0022–3*) showed more transcripts at 3°C. At the same time, Csp gene expression levels and differences tapered off. The gene encoding helicase CsdA (*yptb0486*) was significantly more expressed at 3°C at growth point III, but at 28°C at growth points V and VI. An operon encoding a system that utilizes autoinducer AI-2 showed significantly more transcripts at growth point V.

### Expression of cold shock protein genes

**Figure 6:**
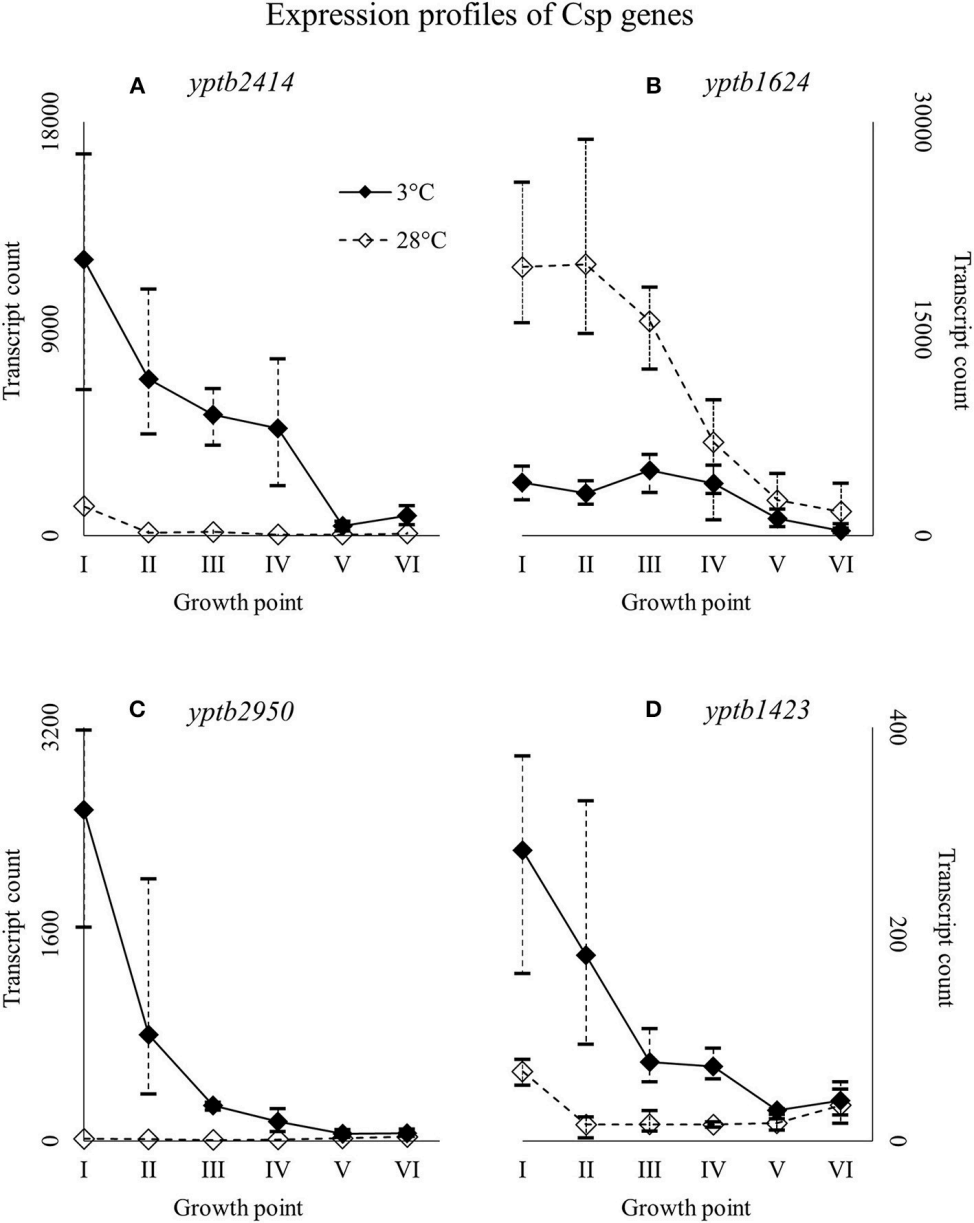
Expression profiles of cold shock protein genes *yptb2414* **(A)***, yptb1624* **(B)**, *yptb2950* **(C)**, and *yptb1423* **(D)** of *Yersinia pseudotuberculosis* IP32953, that was grown at 3 and 28°C and sampled at corresponding growth points I–VI. Median ratio normalized counts were used and the variation between replicates is shown by vertical lines. The expression curves are similar, but *yptb2414, yptb2950*, and *yptb1423* showed significantly more transcripts at 3°C and *yptb1624* at 28°C at the beginning and logarithmic phase of growth.

Of all the Csp genes, *yptb2414* showed most transcripts at 3°C from the beginning of growth throughout the logarithmic growth phase (Figure 6). Its difference in expression between 3 and 28°C was significant at all growth points and the largest of all Csp genes. Gene *yptb1624* was expressed significantly more at 28°C (Figure 6). Gene *yptb2950* showed many more transcripts at 3°C at growth points I–IV, with a peak logFC of over 7 (Figure 6). Its transcription counts dipped after growth point I and decreased toward stationary phase. Gene *yptb1423* was expressed at lower levels, but showed significantly more transcripts at 3°C throughout growth points II–IV (Figure 6).

**Figure 7:**
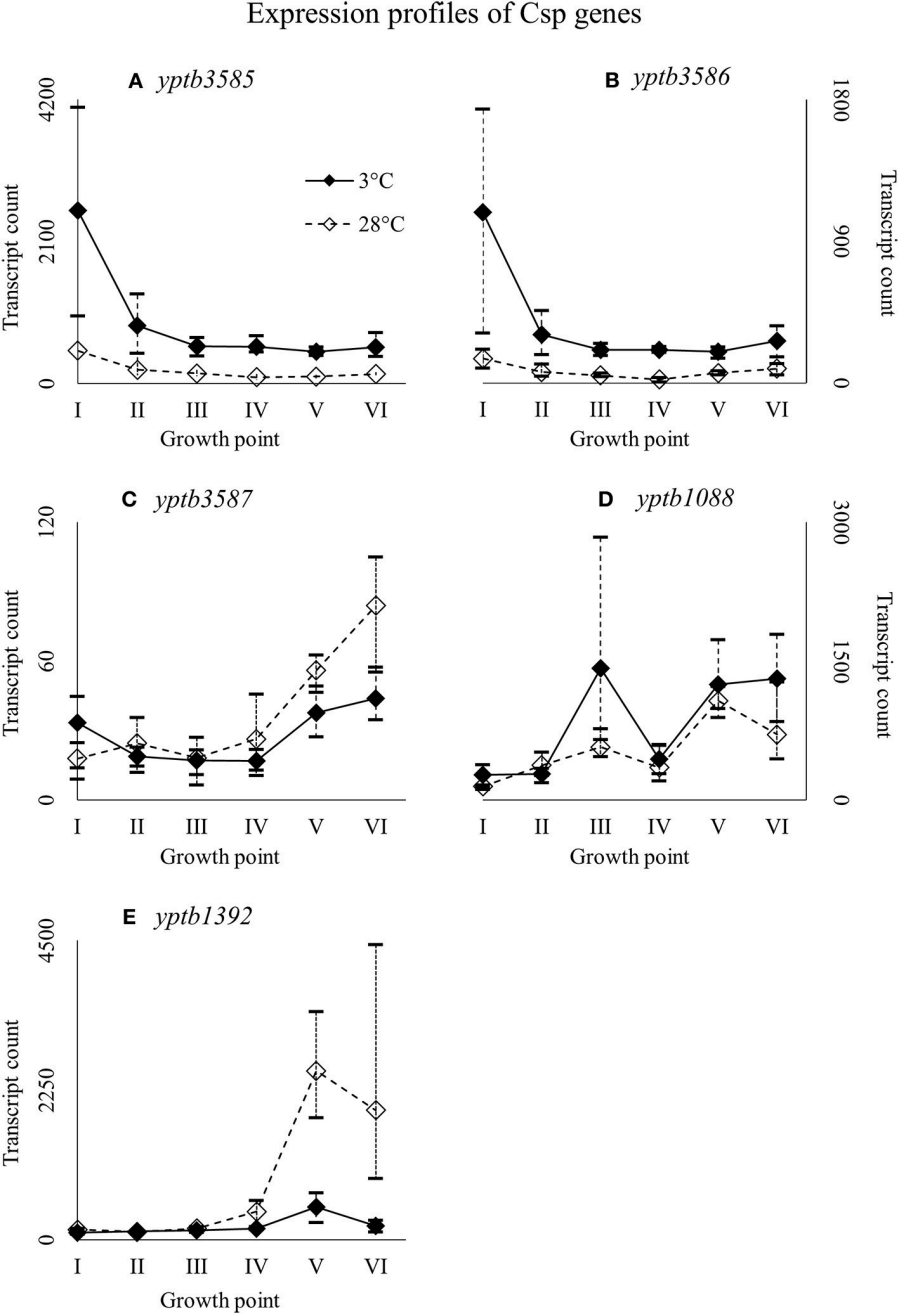
Expression profiles of cold shock protein genes *yptb3585* **(A)***, yptb3586* **(B)**, *yptb3587* **(C)**, *yptb1088* **(D)**, and *yptb1392* **(E)** of *Yersinia pseudotuberculosis* IP32953, that was grown at 3 and 28°C and sampled at corresponding growth points I–VI. Median ratio normalized counts were used and the variation between replicates is shown by vertical lines. Expression of *yptb3585–86* decreased toward stationary phase whereas that of *yptb3687, yptb1088*, and *yptb1392* increased.

Nearly identical genes, *yptb3585* and *yptb3586*, were expressed highly at growth point I, but their expression levels dipped right after (Figure 7). However, the differences in their expression between 3 and 28°C consistently favored cold at significant levels. Expression levels of *yptb3587* and *yptb1088* rose toward the stationary phase at 3°C, but the difference between 3 and 28°C was not significant. After starting growth, *yptb3587* appeared slightly downregulated (Figure 7). Gene *yptb1392* was expressed much in the same way, but showed significantly more transcripts at 28°C at the stationary phase of growth (Figure 7).

### Expression of DEAD-box RNA helicase genes

**Figure 8:**
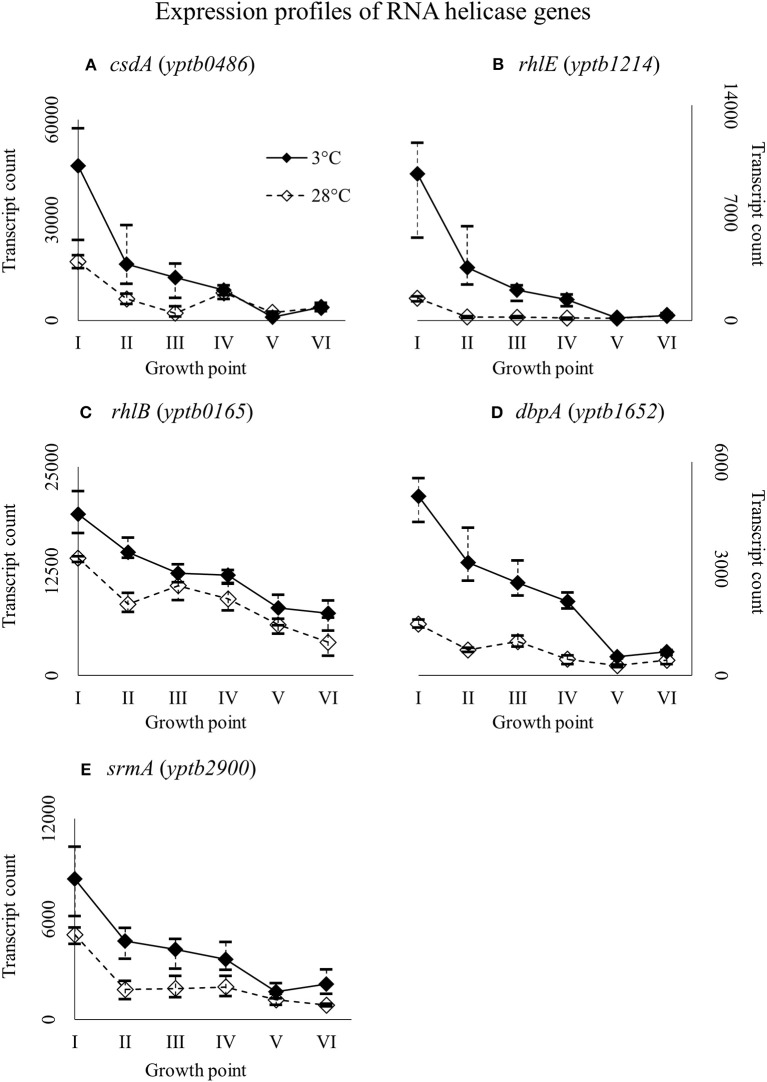
Expression profiles of DEAD-box RNA helicase genes *csdA* **(A)***, rhlE* **(B)**, *rhlB* **(C)**, *dbpA* **(D)**, and *srmA* **(E)** of *Yersinia pseudotuberculosis* IP32953, that was grown at 3 and 28°C and sampled at corresponding growth points I–VI. Median ratio normalized counts were used and the variation between replicates is shown by vertical lines. Gene *csdA* showed significantly more transcripts at growth point III, *rhlE* at growth points I–IV, and *dbpA* at growth points II and IV, although all the genes expressed more at 3°C at least from the beginning through logarithmic phase.

*Y. pseudotuberculosis* has five helicases with a conserved DEAD-box motif: CsdA (*yptb0486*), RhlE (*yptb1214*), RhlB (*yptb0165*), DbpA (*yptb1652*), and SrmA (*yptb2900*). All the helicase genes were expressed more at 3°C throughout growth (Figure 8), but the difference was significant for *csdA* (*yptb0486*) at growth point III, *rhlE* (*yptb1214*) at growth points I–IV, and *dbpA* (*yptb1652*) at growth points II and IV.

### Expression of genes handling compatible solutes

**Figure 9:**
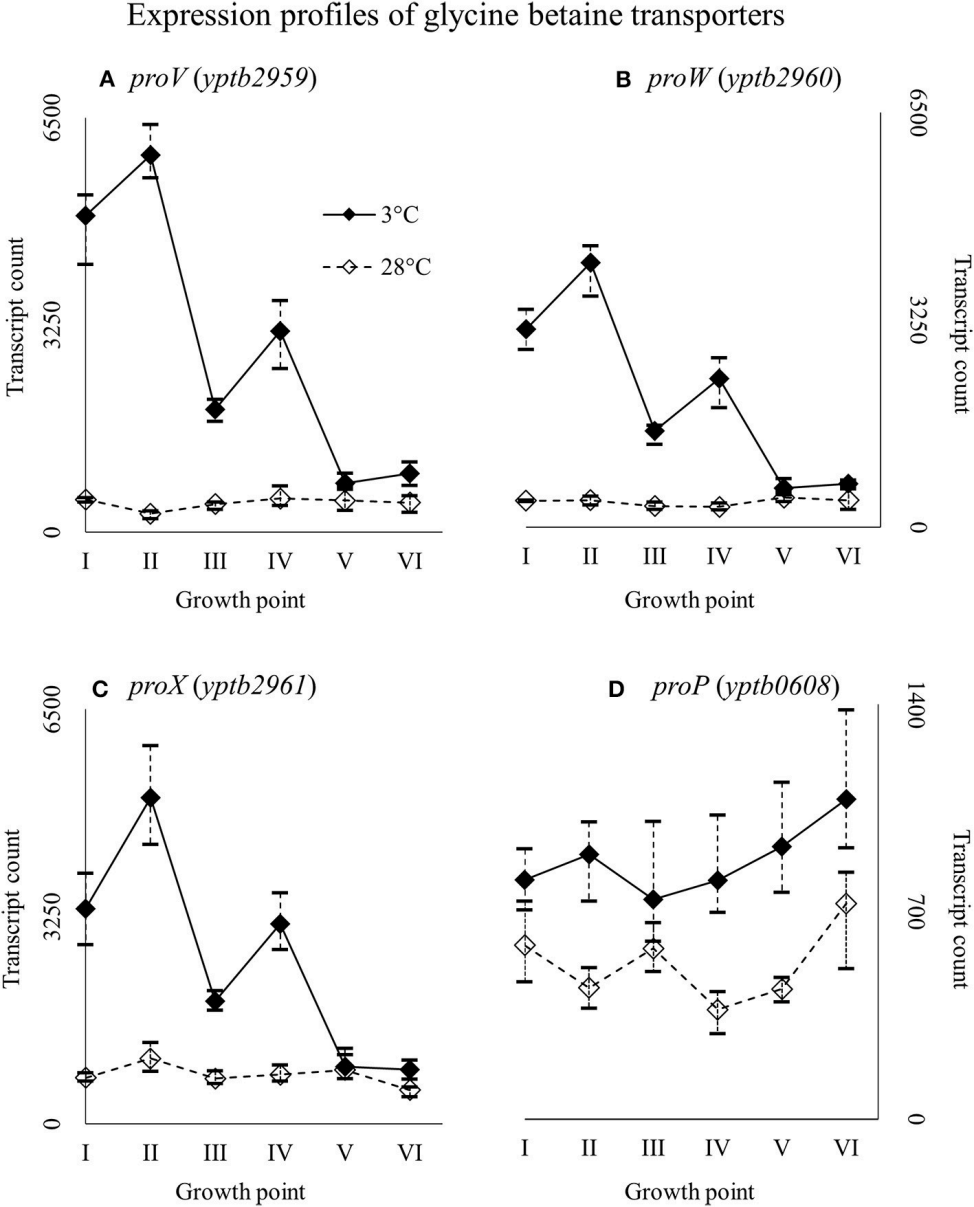
Expression profiles of genes encoding glycine betaine transporters, the *proU* operon (*proVWX*, **A–C)**, and *proP* **(D)** of *Yersinia pseudotuberculosis* IP32953, that was grown at 3 and 28°C and sampled at corresponding growth points I–VI. Median ratio normalized counts were used and the variation between replicates is shown by vertical lines. The *proU* operon is hardly expressed at all at 28°C and *proP* also showed more transcripts at 3°C.

**Figure 10:**
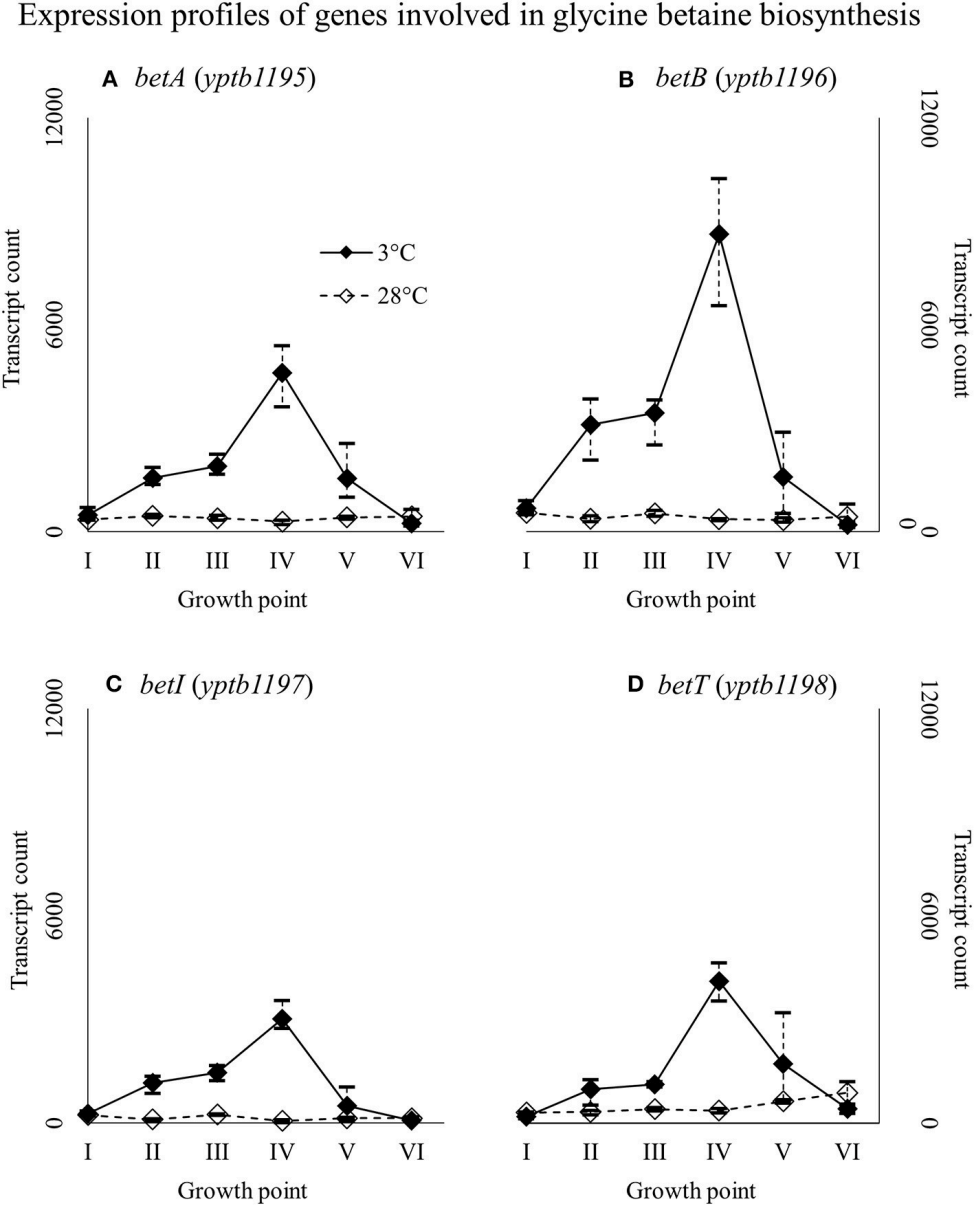
Expression profiles of genes involved in glycine betaine biosynthesis, the *betIBA-betT* divergent operon **(A–D)**, of *Yersinia pseudotuberculosis* IP32953, that was grown at 3 and 28°C and sampled at corresponding growth points I–VI. Median ratio normalized counts were used and the variation between replicates is shown by vertical lines. The expression of the operon is all but diminished at 28°C.

**Figure 11:**
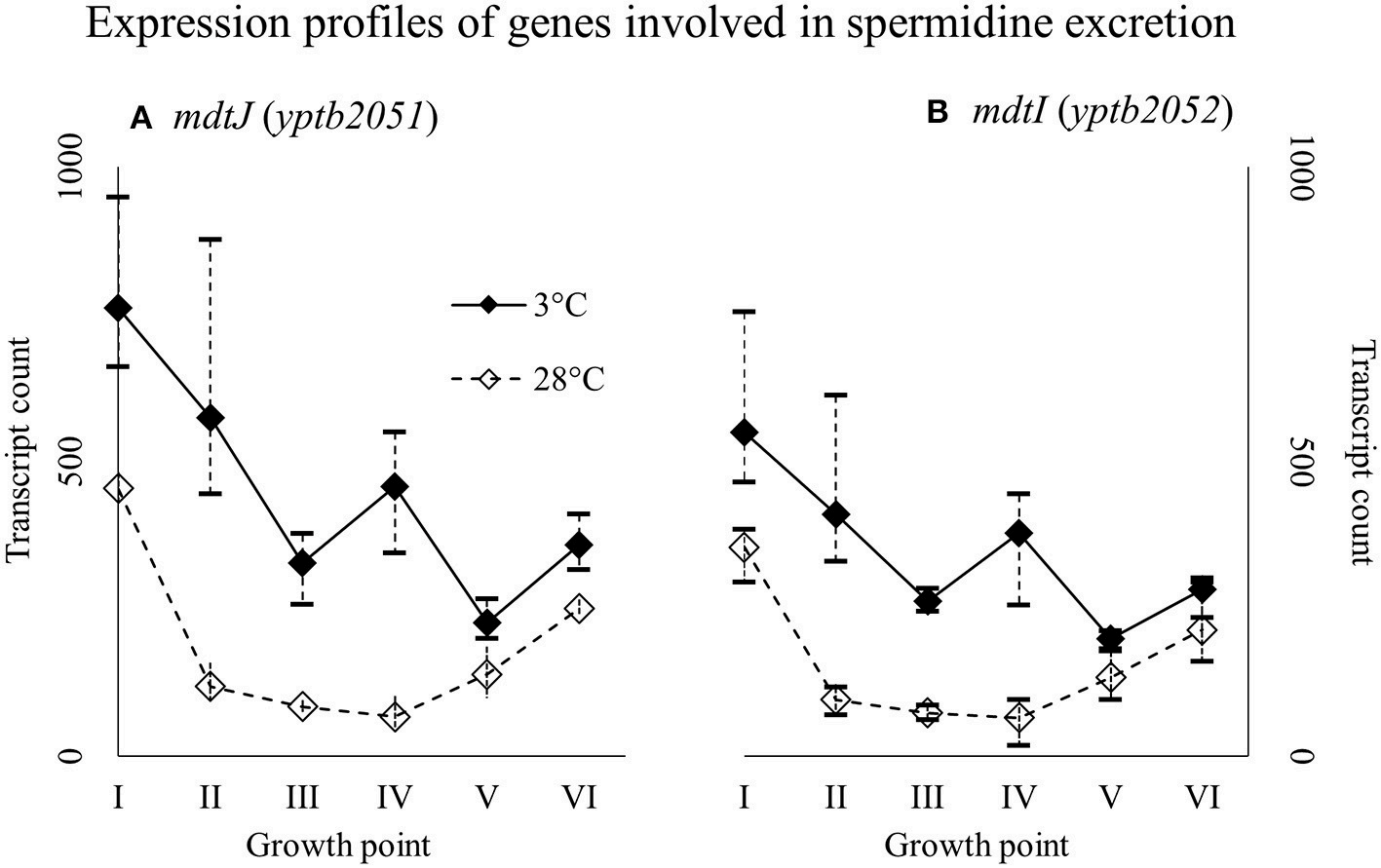
Expression profiles of genes encoding a spermidine efflux pump MdtIJ **(A,B)** of *Yersinia pseudotuberculosis* IP32953, that was grown at 3 and 28°C and sampled at corresponding growth points I–VI. Median ratio normalized counts were used and the variation between replicates is shown by vertical lines. The genes were expressed more at 3°C throughout the growth with a new expression peak at growth point IV.

Genes *yptb2959–61* form the *proU* operon that encodes a transport system for glycine betaine and proline; genes *yptb1195–98* form the *betIBA-betT* divergent operon that is involved in glycine betaine biosynthesis; and genes *yptb2052* and *yptb2051* form the *mdtIJ* operon that encodes a spermidine efflux pump. Operon *proU* showed significantly more transcripts at 3°C at growth points I–IV (Figure 9), operon *betIBA-betT* at growth points II–IV (Figure 10), and operon *mdtIJ* at II and IV (Figure 11). Corresponding functional modules were also overexpressed at growth points I–IV for operon *proU*, points II–V for operon *betIBA-betT*, and points II and IV for operon *mdtIJ* (Figure 4). Another glycine betaine and L-proline transporter encoding gene *proP* (*yptb0608*) showed more transcripts at 3°C throughout the growth, but not quite at significant levels (Figure 9).

### Expression of genes encoding rho factor, IF-1, and RbfA

**Figure 12:**
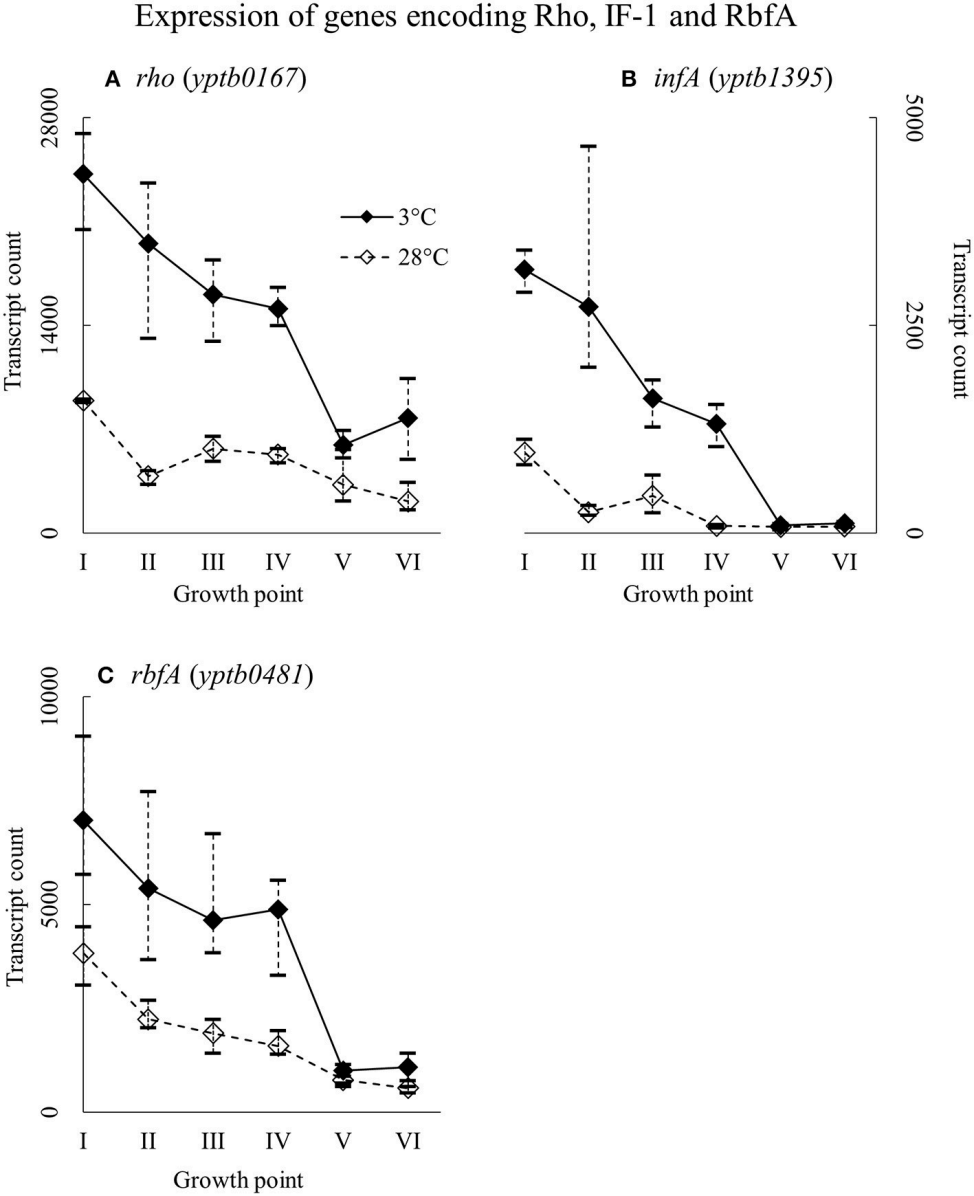
Expression profiles of genes encoding transcription termination factor Rho **(A)**, translation initiation factor IF-1 **(B)**, and ribosome binding factor RbfA **(C)** of *Yersinia pseudotuberculosis* IP32953, that was grown at 3 and 28°C and sampled at corresponding growth points I–VI. Median ratio normalized counts were used and the variation between replicates is shown by vertical lines. All three factors showed more transcripts at 3°C at least to the end of logarithmic phase.

Genes *rho* (*yptb0167*), *infA* (*yptb1395*), and *rbfA* (*yptb0481*) encode the homologs of Rho factor, the translation initiation factor IF-1, and ribosome binding factor RbfA, respectively. The three factor genes were expressed highly from the beginning of growth to the end of the logarithmic phase (Figure 12). Their difference in expression between 3 and 28°C was significant at growth points II and IV, over 16-fold at the largest for *yptb1395*, but the three genes showed more transcripts at 3°C consistently throughout the growth.

### Expression of genes involved in modifying membrane lipid composition

**Figure 13:**
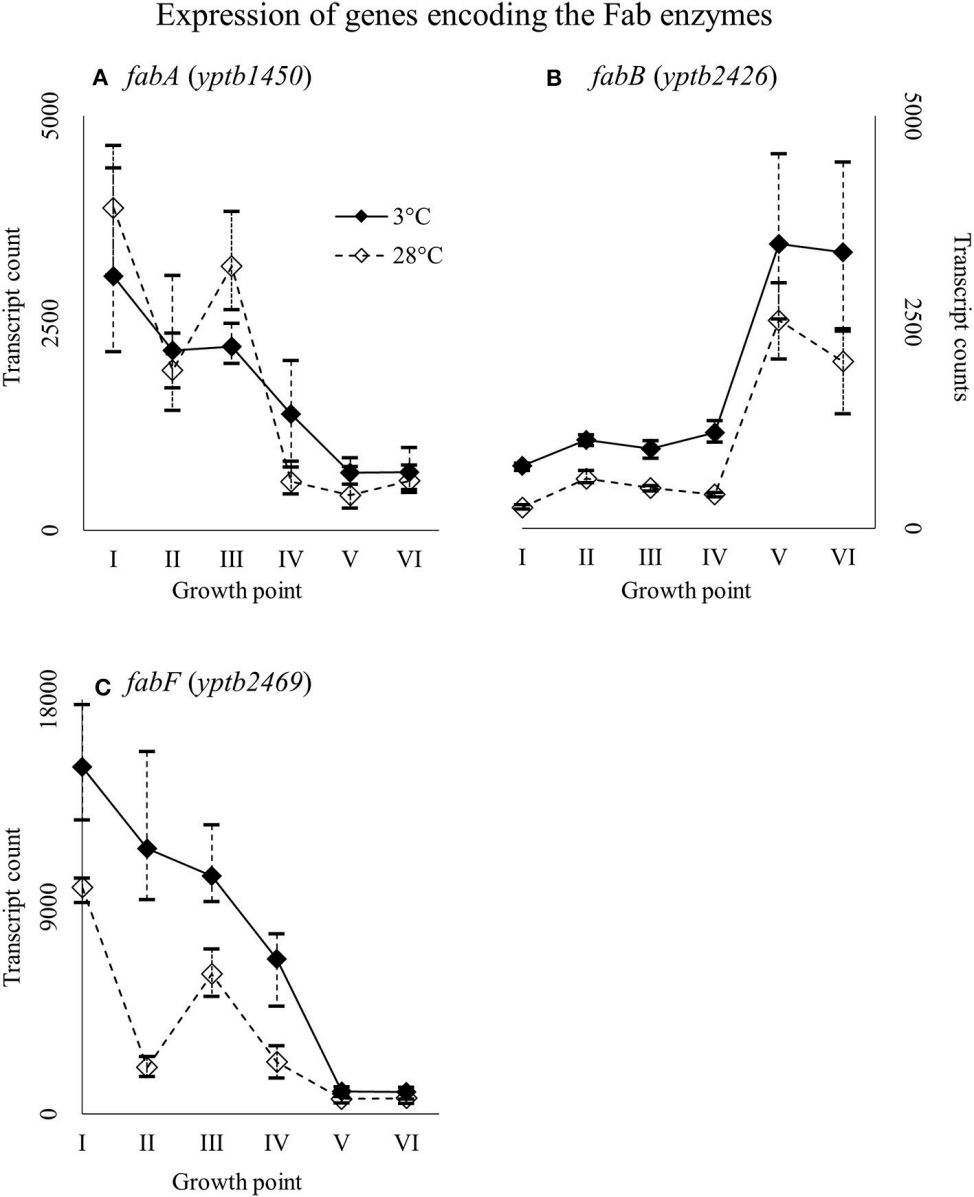
Expression profiles of genes encoding the Fab enzymes *fabA* **(A)**, *fabB* **(B)**, and *fabF* **(C)** of *Yersinia pseudotuberculosis* IP32953, that was grown at 3 and 28°C and sampled at corresponding growth points I–VI. Median ratio normalized counts were used and the variation between replicates is shown by vertical lines. Gene *fabB* and *fabF* showed more transcripts at 3°C during most of the growth.

Genes *yptb1450* and *yptb2469*, encoding FabA and FabF, were expressed highly at the beginning of growth whereas gene *yptb2426* encoding FabB at stationary phase (Figure 13). Gene *yptb2469* showed significantly more transcripts at 3°C at growth point II, although the difference favored cold during most of the growth.

## Discussion

We identified several upregulated genes that may be important to cold adaptation of *Y. pseudotuberculosis*. We also analyzed gene expression profiles to better understand what happens at different phases of growth in cold. To tackle problems posed by refrigerator temperatures, *Y. pseudotuberculosis* has an extensive toolbox that includes Csps, DEAD-box RNA-helicases, compatible solutes, transcription factors, and fatty acid saturases.

### Nutrient acquisition

*Y. pseudotuberculosis* expresses many genes involved in securing nutrients significantly more at 3°C compared to 28°C (Figures 4,5), as successful growth in low temperatures requires more energy. In our results, a shift in nutrient utilization over time is observed. At the beginning of growth, transporters of fructose, ascorbate, maltooligosaccharides, and maltose were upregulated at 3°C. At the beginning of logarithmic phase, transporters of glutamate, aspartate, and arginine were upregulated. Toward the stationary phase, in addition to an operon turning histidine to glutamate, transporters of cystine, methionine, and sulfates were upregulated.

PTSs allow quick acquisition of carbohydrates from the environment (Postma et al., 1993). PTSs have also been linked to regulatory functions during cold stress (Wouters et al., 2000; Monedero et al., 2007). *Y. pseudotuberculosis* expresses several PTSs especially at the beginning of growth significantly more at 3°C than 28°C, as it is in dire need of carbon to sustain its laborious growth. Another large peak in expression of a fructose PTS can be observed at growth point V at 3°C, whereas the peak is located at growth point IV at 28°C. However, this is probably due to slower regulatory processes at 3°C. In *Y. pestis*, the *malMBKEFG*-operon, which is involved in maltose intake, was highly upregulated after cold shock (Han et al., 2005). Maltose can act as an cryoprotectant in addition to being a carbon source (Jain and Roy, 2009). In our results, parts of the *mal*-operon were upregulated especially at the beginning of growth.

*E. coli* has been shown to accumulate certain amino acids, including aspartic acid, glutamic acid, and methionine, when subjected to suboptimal temperatures (Jozefczuk et al., 2010). Accumulation has been theorized to result from protein degradation. In our results, ABC transporters for cystine and methionine were upregulated in cold at the stationary phase.

### Cold shock proteins

*Y. pseudotuberculosis* seems to express Csps encoded by *yptb2414* and *yptb2950* almost exclusively at low temperature, i.e., with the highest significant difference of all Csps, and at high levels. Additionally, *yptb3585, yptb3586*, and *yptb1423* were upregulated throughout the growth. Csps encoded by *yptb2950* and *yptb3585/6* are comparable to CspG and CspB of *E. coli*, respectively (Keto-Timonen et al., 2016). Csp encoded by *yptb2414* is 80% similar to CspC and CspE of *E. coli* K-12 W3110.

Csp encoded by *yptb1088*, which is almost identical to CspE of *E. coli* (Keto-Timonen et al., 2016), showed more transcripts at 3°C, but not at significant levels. However, its transcript count rose toward stationary phase like has been shown to happen in *E. coli* (Czapski and Trun, 2014). In *E. coli*, CspE was also the most abundant of all *csps* after cold shock and present in the bacterium at all times. The expression of *yptb1392*, similar to CspD of *E. coli*, ramped up at stationary phase in 28°C and a similar but less dramatic bump can be seen in expression profile in 3°C. In *E. coli*, CspD is not cold-induced and is expressed mainly at stationary phase (Yamanaka et al., 2001). It is involved in persister cell and biofilm formation (Kim and Wood, 2010).

In *E. coli*, the genes *cspA, cspB, cspE*, and *cspG* all must be deleted to achieve a cold-sensitive phenotype, and the deletion of only one or two genes leads to overexpression of the rest (Xia et al., 2001). In addition, CspC and CspE seem to double as regulatory elements in stress responses (Phadtare and Inouye, 2001; Phadtare et al., 2006). The two proteins have been shown to be required for expression of fructose PTS at suboptimal temperatures (Phadtare et al., 2006). *E. coli* expresses *cspA, cspB*, and *cspG* even when its protein synthesis is inhibited by antimicrobials (Etchegaray and Inouye, 1999). In *Clostridium botulinum*, deletion of *cspB*, or *cspC* leads to a cold-sensitive phenotype, and *cspC* deletion even hinders growth at 37°C (Söderholm et al., 2011). Genes *cspB* and *cspC* also provide the bacterium resistance to NaCl, pH, and ethanol stress (Derman et al., 2015). In *Staphylococcus aureus, cspB* mutation renders the bacterium susceptible to cold and various antimicrobials (Duval et al., 2010). By contrast, CspC has been shown to induce more strongly in response to antimicrobials, hydrogen peroxide, and arsenate than cold (Chanda et al., 2009).

In our experiment, the expression of the genes *yptb3585–86*, comparable to *cspB* of *E. coli* (Keto-Timonen et al., 2016), dipped after the beginning of growth at 3°C but showed more transcripts at 3°C throughout the growth. Pathogenic *Yersinia* have been shown to hold a tandem gene duplication *cspA1/A2* (Neuhaus et al., 1999), which is very nearly identical to genes yptb3585–87 and needs to be downregulated after the initial cold shock for growth to continue (Neuhaus et al., 2000). In *Yersinia enterocolitica, cspA* and *cspB* were the very first genes expressed after cold-shock with their transcript levels decreasing after the beginning (Bresolin et al., 2006). Similarly, *cspA* and *cspB* expression fluctuated accordingly with temperature cycling in *E. coli* (Ivancic et al., 2013). In light of our results, *yptb3585–86* might play an important role in the initial regulation of cold-inducible genes also in *Y. pseudotuberculosis*. Genes *yptb2414* and *yptb2950* seem to be important at low temperature growth considering their differential expression and transcript levels. It is also probable that most Csps of *Y. pseudotuberculosis* are interchangeable. Csp encoded by *yptb1423* is an odd one out, with a similarity of 54% at best to other Csps of *Y. pseudotuberculosis* (Keto-Timonen et al., 2016), with a peak similarity of 50% to the Csps of *E. coli* K-12 W3110.

### DEAD-box RNA helicases

*Y. pseudotuberculosis* is unable to grow at 3°C without a functional RNA helicase gene *csdA* (*yptb0486*) (Palonen et al., 2012). Of other foodborne pathogens, *Listeria monocytogenes* and *C. botulinum* have been shown to require RNA-helicases, including a CsdA homolog, to grow at suboptimal temperatures (Markkula et al., 2012; Söderholm et al., 2015). In fact, CsdA was the most abundant protein extracted from the microbial mats of Lake Joyce in Antarctica (Koo et al., 2016). CsdA and SrmB of *E. coli* are directly involved in the biogenesis of large ribosomal subunits, presumably at subsequent steps with some functional overlap, and deletion of either gene stunts growth at low temperatures (Charollais et al., 2003, 2004). The deletion of *dbpA* in *E. coli* did not lead to similar accumulation of ribosome precursors and stunted growth at either 25 or 37°C (Peil et al., 2008).

Our results suggest that DbpA (encoded by *yptb1652*) may indeed be important at low temperatures, at least in *Y. pseudotuberculosis*. DbpA is missing completely from *Pasteurellales*, an order of symbionts and parasites growing at unchanging, high-temperature, nutrient-rich niches, which suggests that DbpA, like other DEAD-box helicases, has become specialized to operate in adverse growth conditions (Iost and Dreyfus, 2006). Amino acid similarities between corresponding helicases of *Y. pseudotuberculosis* IP32953 and *E. coli* K-12 W3110 range from 69 to 90%.

Gene *yptb1214* coding RhlE had the largest and most consistent significant difference in expression in favor of 3°C. It has been suggested by Jain (2008) that RhlE may modulate the function of CsdA and SrmB in priming immature ribosomal RNA. This would also help explain the gene's prominence at low temperature in our results at the beginning and during logarithmic phase when ribosomal activity is at its highest. In *E. coli*, RhlE has been shown to complement the function of both CsdA and RhlB (Khemici et al., 2004; Awano et al., 2007), but deletion of *rhlE* did not affect growth at 37°C in *E. coli* (Phadtare, 2011).

### Compatible solutes

Our results show that *Y. pseudotuberculosis* expresses genes involved in glycine betaine intake and biosynthesis significantly more at 3°C during critical points of growth, i.e., the beginning and logarithmic phase. It has been shown that *Y. enterocolitica*, with almost identical *proU* and *proP* operons, accumulates glycine betaine at low temperature, but glycine betaine only protects it against osmotic stress (Park et al., 1995; Annamalai and Venkitanarayanan, 2009). However, glycine betaine has been shown to also grant cold protection to gram-positive *L. monocytogenes, B. subtilis*, and gram-negative *Vibrio anguillarum* (Ko et al., 1994; Hoffmann and Bremer, 2011; Ma et al., 2017). Furthermore, choline/carnitine/betaine transporter gene of *C. botulinum* was upregulated in cold shock (Dahlsten et al., 2014). The precise mechanism behind glycine betaine meditated cold protection is unclear, but the molecule has been suggested to hinder ice crystal formation and displace water around macromolecules, reducing aggregation and denaturation (Hoffmann and Bremer, 2011).

In our results, an operon encoding spermidine efflux pump MdtIJ was expressed significantly more at 3°C. Bacteria need spermidine for various purposes, but at low temperatures excess spermidine is detrimental (Higashi et al., 2008). Spermidine displaces magnesium-ions from ribosomes, thus deactivating them, but it also prolongs synthesis of Csps after cold shock (Limsuwun and Jones, 2000). It has been shown that prolonged synthesis of Csps after cold shock occupies all available ribosomes effectively stopping growth in *Y. enterocolitica* (Neuhaus et al., 2000). Our results suggest that also *Y. pseudotuberculosis* ejects excess spermidine to avoid these problems.

### Rho factor, IF-1, and RbfA

Translation factors like IF-1 and RbfA act directly on ribosomes, securing protein synthesis. Initiation factor IF-1 separates the overly stabilized large and small subunits of ribosomes so that they can fuse and begin translating again elsewhere (Giangrossi et al., 2007), while RbfA primes 16S rRNA for new small subunits (Xia et al., 2003). Both IF-1 and RbfA have been shown to be significantly more expressed during cold shock in *E. coli* (Xia et al., 2003; Giangrossi et al., 2007). *E. coli* IF-1 resembles the CspA homologs (Phadtare et al., 2007), whereas in *Y. pseudotuberculosis* they are only 42–50% similar in nucleic acid and 14–21% in amino acid sequence.

The *rho* gene is associated with bacteria that are subjected to various stresses in their environment (D'heygère et al., 2013). Rho factor is needed at low temperature to clear the bacterial DNA of frozen ribosomes and polymerases (D'heygère et al., 2013), and the structure of its RNA-binding subunit is similar to that of a cold shock domain (Briercheck et al., 1996). Rho factor also coordinates homeostasis of magnesium ions, which are needed by ribosomes, by controlling transporter genes (Kriner and Groisman, 2015). The Rho factor has been shown to be upregulated at low temperature in at least *Acidithiobacillus ferrooxidans* (Mykytczuk et al., 2011), *Pseudomonas haloplanktis* strain TAC125 (Piette et al., 2010), and *Bacillus subtilis* (Quirk et al., 1993). To our knowledge, none of these factors have been linked to cold growth in *Yersinia* genus before.

*Y. pseudotuberculosis* expressed the genes encoding IF-1, RbfA, and Rho all through the critical phases of growth at high levels but also significantly more at 3°C. Massive difference of IF-1 (*yptb1395*) expression in favor of cold during logarithmic phase suggests that the initiation factor is important for continued growth at low temperature. It is probable that IF-1 and RbfA of *Y. pseudotuberculosis* provide the bacterium with working ribosomes at low temperature. *Y. pseudotuberculosis* might need Rho factor not only to terminate inefficient transcription but also to provide ribosomes with much needed magnesium ions. The upregulation of *mgt* transporter genes at 3°C throughout the growth supports this theory.

### Cell membranes

Lipid synthesis and cell growth cease in cyanobacteria until an adequate membrane lipid composition has been achieved (Sinetova and Los, 2016). However, gram-negative cyanobacteria and gram-positive *Bacillus subtilis* possess a two-component system, DesK-DesR, that senses increasing membrane rigidity at low temperatures (Beranová et al., 2010; Sinetova and Los, 2016). Although a corresponding two-component system has not been identified in *Y. pseudotuberculosis* (Palonen et al., 2011), the ratio of unsaturated to saturated fatty acids in its membranes rises at low temperature (Bakholdina et al., 2004). In another gram-negative bacterium, *E. coli*, temperature drop directly affects activity of the FabF enzyme in its cytosol and this enzyme is involved in unsaturated lipid synthesis with FabA and FabB (Mansilla et al., 2004). Cold stress has also been shown to induce the transcription of *fabF* in *Y. pestis* (Han et al., 2005).

It is also possible that the csp encoded by *yptb2414*, a homolog of *E. coli* CspC and CspE, is involved in cold adaptation of membranes in *Y. pseudotuberculosis*. CspC and CspE bind specific uracil-rich segments of mRNA, and these segments often encode hydrophobic structures of membrane proteins (Benhalevy et al., 2015). It has been suggested that bacteria use these segments as addresses for directing membrane protein mRNA to where they are needed in the cell (Benhalevy et al., 2015).

## Conclusions

*Y. pseudotuberculosis* has extensive tools to keep its protein synthesis running at low temperature. It is probable, that the functions of its Csps, RNA helicases and factors acting on ribosomes overlap greatly and thus form a robust network to protect nucleic acids from cold damage. Csps encoded by *yptb1423, yptb2414, yptb2950, yptb3585–86*, and RNA helicases CsdA, RhlE, and DbpA, seem to form the backbone of cold survival of *Y. pseudotuberculosis* with their regulatory and nucleic acid unwinding functions. IF-1, RbfA, and by extension, Rho, keep the ribosomes of *Y. pseudotuberculosis* running even at suboptimal temperatures. Rho factor also terminates frozen ribosomes and RNA polymerases, freeing scant resources under cold stress.

Increased expression of motility, chemotaxis, and nutrient uptake genes at low temperature suggests that *Y. pseudotuberculosis* actively tries to find and secure sufficient resources to grow in refrigerator temperatures. The nutrients acquired are diverse and the nutrient profile seems to shift as growth progresses. The bacterium also possibly changes its membrane lipid composition with Fab enzymes, much like *E. coli*, to battle increasing membrane rigidity.

The exact role of glycine betaine in cold resistance is uncertain, but *Y. pseudotuberculosis* seems to accumulate it during cold growth like many other bacteria. Genes involved in defense against foreign DNA as well as oxidative stress were upregulated at 3°C, which would seem to suggest that stress responses are linked in some way.

## Author contributions

RK-T and HK designed the study. NS and RK-T performed the experiments. J-PV and KJ performed the transcriptome analysis. J-PV, KJ, RK-T, and HK contributed to the data analysis and interpretation. J-PV drafted the manuscript. RK-T, NS, and HK contributed to manuscript revision. All authors have read and approved the final manuscript.

### Conflict of interest statement

The authors declare that the research was conducted in the absence of any commercial or financial relationships that could be construed as a potential conflict of interest.

## References

[B1] Annamalai T. Venkitanarayanan K. (2009). Role of *proP* and *proU* in betaine uptake by *Yersinia enterocolitica* under cold and osmotic stress conditions. Appl. Environ. Microbiol. 75, 1471–1477. 10.1128/AEM.01644-08 19114512 PMC2655450

[B2] Awano N. Xu C. Ke H. Inoue K. Inouye M. Phadtare S. (2007). Complementation analysis of the cold-sensitive phenotype of the *Escherichia coli csdA* deletion strain. J. Bacteriol. 189, 5808–5815. 10.1128/JB.00655-07 17557820 PMC1952031

[B3] Bakholdina S. I. Sanina N. M. Krasikova I. N. Popova O. B. Solov'eva T. F. (2004). The impact of abiotic factors (temperature and glucose) on physicochemical properties of lipids from *Yersinia pseudotuberculosis*. Biochimie 86, 875–881. 10.1016/j.biochi.2004.10.011 15667937

[B4] Benhalevy D. Bochkareva E. S. Biran I. Bibi E. (2015). Model uracil-rich RNAs and membrane protein mRNAs interact specifically with cold shock proteins in *Escherichia coli*. PLoS ONE 10:e0134413. 10.1371/journal.pone.0134413 26225847 PMC4520561

[B5] Benjamini Y. Hochberg Y. (1995). Controlling the false discovery rate: a practical and powerful approach to multiple testing. J. R. Stat. Soc. Ser. B Methodol. 57, 289–300.

[B6] Beranová J. Mansilla M. C. De Mendoza D. Elhottová D. Konopásek I. (2010). Differences in cold adaptation of *Bacillus subtilis* under anaerobic and aerobic conditions. J. Bacteriol. 192, 4164–4171. 10.1128/JB.00384-10 20581210 PMC2916416

[B7] Bresolin G. Neuhaus K. Scherer S. Fuchs T. M. (2006). Transcriptional analysis of long-term adaptation of *Yersinia enterocolitica* to low-temperature growth. J. Bacteriol. 188, 2945–2958. 10.1128/JB.188.8.2945-2958.2006 16585756 PMC1447024

[B8] Briercheck D. M. Allison T. J. Richardson J. P. Ellena J. F. Wood T. C. Rule G. S. (1996). 1H, 15N and 13C resonance assignments and secondary structure determination of the RNA-binding domain of *E. coli* Rho protein. J. Biomol. NMR 8, 429–444. 10.1007/BF00228145 9008362

[B9] Buzoleva L. S. Somov G. P. (2003). Adaptation variability of *Yersinia pseudotuberculosis* during long-term persistence in soil. Bull. Exp. Biol. Med. 135, 456–459. 10.1023/A:1024915409187 12910285

[B10] Chanda P. K. Mondal R. Sau K. Sau S. (2009). Antibiotics, arsenate and H2O2 induce the promoter of *Staphylococcus aureus* cspC gene more strongly than cold. J. Basic Microbiol. 49, 205–211. 10.1002/jobm.200800065 18803257

[B11] Charollais J. Dreyfus M. Iost I. (2004). CsdA, a cold-shock RNA helicase from *Escherichia coli*, is involved in the biogenesis of 50S ribosomal subunit. Nucleic Acids Res. 32, 2751–2759. 10.1093/nar/gkh603 15148362 PMC419605

[B12] Charollais J. Pflieger D. Vinh J. Dreyfus M. Iost I. (2003). The DEAD-box RNA helicase SrmB is involved in the assembly of 50S ribosomal subunits in *Escherichia coli*. Mol. Microbiol. 48, 1253–1265. 10.1046/j.1365-2958.2003.03513.x 12787353

[B13] Chattopadhyay M. K. Raghu G. Sharma Y. V. R. K. Biju A. R. Rajasekharan M. V. Shivaji S. (2011). Increase in oxidative stress at low temperature in an antarctic bacterium. Curr. Microbiol. 62, 544–546. 10.1007/s00284-010-9742-y 20730433

[B14] Czapski T. R. Trun N. (2014). Expression of csp genes in *E. coli* K-12 in defined rich and defined minimal media during normal growth, and after cold-shock. Gene 547, 91–97. 10.1016/j.gene.2014.06.033 24952137

[B15] Dahlsten E. Isokallio M. Somervuo P. Lindström M. Korkeala H. (2014). Transcriptomic analysis of (Group I) *Clostridium botulinum* ATCC 3502 cold shock response. PLoS ONE 9:e89958. 10.1371/journal.pone.0089958 24587151 PMC3933689

[B16] Derman Y. Söderholm H. Lindström M. Korkeala H. (2015). Role of csp genes in NaCl, pH, and ethanol stress response and motility in *Clostridium botulinum* ATCC 3502. Food Microbiol. 46, 463–470. 10.1016/j.fm.2014.09.004 25475316

[B17] D'heygère F. Rabhi M. Boudvillain M. (2013). Phyletic distribution and conservation of the bacterial transcription termination factor Rho. Microbiology 159, 1423–1436. 23704790 10.1099/mic.0.067462-0

[B18] Duval B. D. Mathew A. Satola S. W. Shafer W. M. (2010). Altered growth, pigmentation, and antimicrobial susceptibility properties of *Staphylococcus aureus* due to loss of the major cold shock gene *cspB*. Antimicrob. Agents Chemother. 54, 2283–2290. 10.1128/AAC.01786-09 20368405 PMC2876397

[B19] Etchegaray J. P. Inouye M. (1999). CspA, CspB, and CspG, major cold shock proteins of *Escherichia coli*, are induced at low temperature under conditions that completely block protein synthesis. J. Bacteriol. 181, 1827–1830. 10074075 10.1128/jb.181.6.1827-1830.1999 PMC93581

[B20] Gaidatzis D. Lerch A. Hahne F. Stadler M. B. (2015). QuasR: quantification and annotation of short reads in R. Bioinformatics 31, 1130–1132. 10.1093/bioinformatics/btu781 25417205 PMC4382904

[B21] Gengler S. Laudisoit A. Batoko H. Wattiau P. (2015). Long-Term Persistence of *Yersinia pseudotuberculosis* in entomopathogenic nematodes. PLoS ONE 10:e0116818. 10.1371/journal.pone.0116818 25635766 PMC4312075

[B22] Giangrossi M. Brandi A. Giuliodori A. M. Gualerzi C. O. Pon C. L. (2007). Cold-shock-induced *de novo* transcription and translation of *infA* and role of IF1 during cold adaptation. Mol. Microbiol. 64, 807–821. 10.1111/j.1365-2958.2007.05699.x 17462025

[B23] Giannitti F. Barr B. C. Brito B. P. Uzal F. A. Villanueva M. Anderson M. (2014). *Yersinia pseudotuberculosis* infections in goats and other animals diagnosed at the California animal health and food safety laboratory system: 1990–2012. J. Vet. Diagn. Invest. 26, 88–95. 10.1177/1040638713516624 24442485

[B24] Han Y. Zhou D. Pang X. Zhang L. Song Y. Tong Z.. (2005). DNA microarray analysis of the heat- and cold-shock stimulons in *Yersinia pestis*. Microbes Infect. 7, 335–348. 10.1016/j.micinf.2004.11.005 15777740

[B25] Hardcastle T. J. Kelly K. A. (2010). baySeq: Empirical Bayesian methods for identifying differential expression in sequence count data. BMC Bioinformatics 11:422. 10.1186/1471-2105-11-422 20698981 PMC2928208

[B26] Higashi K. Ishigure H. Demizu R. Uemura T. Nishino K. Yamaguchi A.. (2008). Identification of a spermidine excretion protein complex (MdtJI) in *Escherichia coli*. J. Bacteriol. 190, 872–878. 10.1128/JB.01505-07 18039771 PMC2223573

[B27] Hoffmann T. Bremer E. (2011). Protection of *Bacillus subtilis* against cold stress via compatible-solute acquisition. J. Bacteriol. 193, 1552–1562. 10.1128/JB.01319-10 21296969 PMC3067655

[B28] Huber W. Carey V. J. Gentleman R. Anders S. Carlson M. Carvalho B. S.. (2015). Orchestrating high-throughput genomic analysis with Bioconductor. Nat. Methods 12:115. 10.1038/nmeth.3252 25633503 PMC4509590

[B29] Iost I. Dreyfus M. (2006). DEAD-box RNA helicases in *Escherichia coli*. Nucl. Acids Res. 34, 4189–4197. 10.1093/nar/gkl500 16935881 PMC1616957

[B30] Ivancic T. Jamnik P. Stopar D. (2013). Cold shock CspA and CspB protein production during periodic temperature cycling in *Escherichia coli*. BMC Res. Notes 6:248. 10.1186/1756-0500-6-248 23815967 PMC3704898

[B31] Jaakkola K. Somervuo P. Korkeala H. (2015). Comparative genomic hybridization analysis of *Yersinia enterocolitica* and *Yersinia pseudotuberculosis* identifies genetic traits to elucidate their different ecologies. BioMed. Res. Int. 2015:760494. 10.1155/2015/760494 26605338 PMC4641178

[B32] Jain C. (2008). The *E. coli* RhlE RNA helicase regulates the function of related RNA helicases during ribosome assembly. RNA 14, 381–389. 10.1261/rna.800308 18083833 PMC2212244

[B33] Jain N. K. Roy I. (2009). Effect of trehalose on protein structure. Protein Sci. 18, 24–36. 10.1002/pro.3 19177348 PMC2708026

[B34] Johnson S. L. Daligault H. E. Davenport K. W. Jaissle J. Frey K. G. Ladner J. T.. (2015). Thirty-two complete genome assemblies of nine *Yersinia* species, including *Y*. pestis, Y. pseudotuberculosis, and Y. enterocolitica. Genome Announc. 3:e00148–15. 10.1128/genomeA.00148-15 25931590 PMC4417686

[B35] Joutsen S. Laukkanen-Ninios R. Henttonen H. Niemimaa J. Voutilainen L. Kallio E. R.. (2017). *Yersinia* spp. in Wild Rodents and Shrews in Finland. Vector-Borne Zoonotic Dis. 17, 303–311. 10.1089/vbz.2016.2025 28332937

[B36] Jozefczuk S. Klie S. Catchpole G. Szymanski J. Cuadros-Inostroza A. Steinhauser D.. (2010). Metabolomic and transcriptomic stress response of *Escherichia coli*. Mol. Syst. Biol. 6:364. 10.1038/msb.2010.18 20461071 PMC2890322

[B37] Kanehisa M. Goto S. (2000). KEGG: kyoto encyclopedia of genes and genomes. Nucleic Acids Res. 28, 27–30. 10.1093/nar/28.1.27 10592173 PMC102409

[B38] Keto-Timonen R. Hietala N. Palonen E. Hakakorpi A. Lindström M. Korkeala H. (2016). Cold shock proteins: a minireview with special emphasis on csp-family of enteropathogenic *Yersinia*. Front. Microbiol. 7:1151. 10.3389/fmicb.2016.01151 27499753 PMC4956666

[B39] Keto-Timonen R. Pontinen A. Aalto-Araneda M. Korkeala H. (2018). Growth of *Yersinia pseudotuberculosis* strains at different temperatures, pH values, and NaCl and ethanol concentrations. J. Food Prot. 81, 142–149. 10.4315/0362-028X.JFP-17-223 29283703

[B40] Khemici V. Toesca I. Poljak L. Vanzo N. F. Carpousis A. J. (2004). The RNase E of *Escherichia coli* has at least two binding sites for DEAD-box RNA helicases: functional replacement of RhlB by RhlE. Mol. Microbiol. 54, 1422–1430. 10.1111/j.1365-2958.2004.04361.x 15554979

[B41] Kim Y. Wood T. K. (2010). Toxins Hha and CspD and small RNA regulator Hfq are involved in persister cell formation through MqsR in *Escherichia coli*. Biochem. Biophys. Res. Commun. 391, 209–213. 10.1016/j.bbrc.2009.11.033 19909729 PMC2812665

[B42] Ko R. Smith L. T. Smith G. M. (1994). Glycine betaine confers enhanced osmotolerance and cryotolerance on *Listeria monocytogenes*. J. Bacteriol. 176, 426–431. 10.1128/jb.176.2.426-431.1994 8288538 PMC205066

[B43] Koo H. Hakim J. A. Fisher P. R. E. Grueneberg A. Andersen D. T. Bej A. K. (2016). Distribution of cold adaptation proteins in microbial mats in Lake Joyce, Antarctica: analysis of metagenomic data by using two bioinformatics tools. J. Microbiol. Methods 120, 23–28. 10.1016/j.mimet.2015.11.008 26578243

[B44] Kriner M. A. Groisman E. A. (2015). The bacterial transcription termination factor Rho coordinates Mg2+ homeostasis with translational signals. J. Mol. Biol. 427, 3834–3849. 10.1016/j.jmb.2015.10.020 26523680 PMC4964609

[B45] Laukkanen R. Martínez P. O. Siekkinen K.-M. Ranta J. Maijala R. Korkeala H. (2008). Transmission of *Yersinia pseudotuberculosis* in the Pork Production Chain from Farm to Slaughterhouse. Appl. Environ. Microbiol. 74, 5444–5450. 10.1128/AEM.02664-07 18641149 PMC2546633

[B46] Le Guern A.-S. Martin L. Savin C. Carniel E. (2016). Yersiniosis in France: overview and potential sources of infection. Int. J. Infect. Dis. 46, 1–7. 10.1016/j.ijid.2016.03.008 26987478

[B47] Limsuwun K. Jones P. G. (2000). Spermidine acetyltransferase is required to prevent spermidine toxicity at low temperatures in *Escherichia coli*. J. Bacteriol. 182, 5373–5380. 10.1128/JB.182.19.5373-5380.2000 10986239 PMC110979

[B48] Love M. I. Huber W. Anders S. (2014). Moderated estimation of fold change and dispersion for RNA-seq data with DESeq2. Genome Biol. 15:550. 10.1186/s13059-014-0550-8 25516281 PMC4302049

[B49] Ma Y. Wang Q. Gao X. Zhang Y. (2017). Biosynthesis and uptake of glycine betaine as cold-stress response to low temperature in fish pathogen *Vibrio anguillarum*. J. Microbiol. 55, 44–55. 10.1007/s12275-017-6370-2 28035596

[B50] Mansilla M. C. Cybulski L. E. Albanesi D. De Mendoza D. (2004). Control of membrane lipid fluidity by molecular thermosensors. J. Bacteriol. 186, 6681–6688. 10.1128/JB.186.20.6681-6688.2004 15466018 PMC522199

[B51] Markkula A. Mattila M. Lindström M. Korkeala H. (2012). Genes encoding putative DEAD-box RNA helicases in *Listeria monocytogenes* EGD-e are needed for growth and motility at 3°C. Environ. Microbiol. 14, 2223–2232. 10.1111/j.1462-2920.2012.02761.x 22564273

[B52] Monedero V. Maze A. Boel G. Zuniga M. Beaufils S. Hartke A.. (2007). The phosphotransferase system of *Lactobacillus casei*: regulation of carbon metabolism and connection to cold shock response. J. Mol. Microbiol. Biotechnol. 12, 20–32. 10.1159/000096456 17183208

[B53] Mykytczuk N. C. S. Trevors J. T. Foote S. J. Leduc L. G. Ferroni G. D. Twine S. M. (2011). Proteomic insights into cold adaptation of psychrotrophic and mesophilic *Acidithiobacillus ferrooxidans* strains. Antonie van Leeuwenhoek 100, 259–277. 10.1007/s10482-011-9584-z 21604047

[B54] Neuhaus K. Francis K. P. Rapposch S. Görg A. Scherer S. (1999). Pathogenic *Yersinia* species carry a novel, cold-inducible major cold shock protein tandem gene duplication producing both bicistronic and monocistronic mRNA. J. Bacteriol. 181, 6449–6455. 10515936 10.1128/jb.181.20.6449-6455.1999 PMC103781

[B55] Neuhaus K. Rapposch S. Francis K. P. Scherer S. (2000). Restart of exponential growth of cold-shocked *Yersinia enterocolitica* occurs after down-regulation of *cspA1/A2* mRNA. J. Bacteriol. 182, 3285–3288. 10.1128/JB.182.11.3285-3288.2000 10809713 PMC94520

[B56] Niskanen T. Waldenström J. Fredriksson-Ahomaa M. Olsen B. Korkeala H. (2003). *virF*-Positive *Yersinia pseudotuberculosis* and *Yersinia enterocolitica* found in migratory birds in Sweden. Appl. Environ. Microbiol. 69, 4670–4675. 10.1128/AEM.69.8.4670-4675.2003 12902256 PMC169077

[B57] Nuorti J. P. Niskanen T. Hallanvuo S. Mikkola J. Kela E. Hatakka M.. (2004). A widespread outbreak of *Yersinia pseudotuberculosis* O:3 infection from iceberg lettuce. J. Infect. Dis. 189, 766–774. 10.1086/381766 14976592

[B58] Palonen E. Lindström M. Karttunen R. Somervuo P. Korkeala H. (2011). Expression of signal transduction system encoding genes of *Yersinia pseudotuberculosis* IP32953 at 28°C and 3°C. PLoS ONE 6:e25063. 10.1371/journal.pone.0025063 21949852 PMC3176822

[B59] Palonen E. Lindström M. Korkeala H. (2010). Adaptation of enteropathogenic Yersinia to low growth temperature. Crit. Rev. Microbiol. 36, 54–67. 10.3109/10408410903382581 20088683

[B60] Palonen E. Lindström M. Somervuo P. Johansson P. Björkroth J. Korkeala H. (2012). Requirement for RNA Helicase CsdA for Growth of *Yersinia pseudotuberculosis* IP32953 at low temperatures. Appl. Environ. Microbiol. 78, 1298–1301. 10.1128/AEM.07278-11 22156424 PMC3273003

[B61] Park S. Smith L. T. Smith G. M. (1995). Role of glycine betaine and related osmolytes in osmotic stress adaptation in *Yersinia enterocolitica* ATCC 9610. Appl. Environ. Microbiol. 61, 4378–4381. 16535192 10.1128/aem.61.12.4378-4381.1995 PMC1388657

[B62] Pärn T. Hallanvuo S. Salmenlinna S. Pihlajasaari A. Heikkinen S. Telkki-Nykänen H.. (2015). Outbreak of *Yersinia pseudotuberculosis* O:1 infection associated with raw milk consumption, Finland, spring 2014. Eurosurveillance 20:30033. 10.2807/1560-7917.ES.2015.20.40.30033 26537540

[B63] Peil L. Virumäe K. Remme J. (2008). Ribosome assembly in *Escherichia coli* strains lacking the RNA helicase DeaD/CsdA or DbpA. FEBS J. 275, 3772–3782. 10.1111/j.1742-4658.2008.06523.x 18565105

[B64] Pfaffl M. W. (2001). A new mathematical model for relative quantification in real-time RT-PCR. Nucleic Acids Res. 29:e45. 10.1093/nar/29.9.e45 11328886 PMC55695

[B65] Phadtare S. (2011). Unwinding activity of cold shock proteins and RNA metabolism. RNA Biol. 8, 394–397. 10.4161/rna.8.3.14823 21445001 PMC3218510

[B66] Phadtare S. Inouye M. (2001). Role of CspC and CspE in regulation of expression of RpoS and UspA, the stress response proteins in *Escherichia coli*. J. Bacteriol. 183, 1205–1214. 10.1128/JB.183.4.1205-1214.2001 11157932 PMC94993

[B67] Phadtare S. Kazakov T. Bubunenko M. Court D. L. Pestova T. Severinov K. (2007). Transcription antitermination by translation initiation factor IF1. J. Bacteriol. 189, 4087–4093. 10.1128/JB.00188-07 17384193 PMC1913383

[B68] Phadtare S. Tadigotla V. Shin W.-H. Sengupta A. Severinov K. (2006). Analysis of *Escherichia coli* global gene expression profiles in response to overexpression and deletion of CspC and CspE. J. Bacteriol. 188, 2521–2527. 10.1128/JB.188.7.2521-2527.2006 16547039 PMC1428408

[B69] Piette F. D'amico S. Struvay C. Mazzucchelli G. Renaut J. Tutino M. L.. (2010). Proteomics of life at low temperatures: trigger factor is the primary chaperone in the Antarctic bacterium *Pseudoalteromonas haloplanktis* TAC125. Mol. Microbiol. 76, 120–132. 10.1111/j.1365-2958.2010.07084.x 20199592

[B70] Postma P. W. Lengeler J. W. Jacobson G. R. (1993). Phosphoenolpyruvate: carbohydrate phosphotransferase systems of bacteria. Microbiol. Rev. 57, 543–594. 8246840 10.1128/mr.57.3.543-594.1993 PMC372926

[B71] Quirk P. G. Dunkley E. A. Lee P. Krulwich T. A. (1993). Identification of a putative *Bacillus subtilis rho* gene. J. Bacteriol. 175, 647–654. 10.1128/jb.175.3.647-654.1993 8423140 PMC196201

[B72] Rimhanen-Finne R. Niskanen T. Hallanvuo S. Makary P. Haukka K. Pajunen S.. (2009). *Yersinia pseudotuberculosis* causing a large outbreak associated with carrots in Finland, 2006. Epidemiol. Infect. 137, 342–347. 10.1017/S0950268807000155 18177523

[B73] Santos-Montañez J. Benavides-Montano J. A. Hinz A. K. Vadyvaloo V. (2015). *Yersinia pseudotuberculosis* IP32953 survives and replicates in trophozoites and persists in cysts of *Acanthamoeba castellanii*. FEMS Microbiol. Lett. 362:fnv091. 10.1093/femsle/fnv091 26025069 PMC4661780

[B74] Sato K. Komazawa M. (1991). *Yersinia pseudotuberculosis* infection in children due to untreated drinking water. Contrib. Microbiol. Immunol. 12, 5–10. 1935113

[B75] Sievers F. Wilm A. Dineen D. Gibson T. J. Karplus K. Li W.. (2011). Fast, scalable generation of high-quality protein multiple sequence alignments using Clustal Omega. Mol. Syst. Biol. 7:539. 10.1038/msb.2011.75 21988835 PMC3261699

[B76] Sinetova M. A. Los D. A. (2016). New insights in cyanobacterial cold stress responses: Genes, sensors, and molecular triggers. Biochim. Biophys. Acta 1860, 2391–2403. 10.1016/j.bbagen.2016.07.006 27422804

[B77] Söderholm H. Derman Y. Lindström M. Korkeala H. (2015). Functional csdA is needed for effective adaptation and initiation of growth of *Clostridium botulinum* ATCC 3502 at suboptimal temperature. Int. J. Food Microbiol. 208, 51–57. 10.1016/j.ijfoodmicro.2015.05.013 26057109

[B78] Söderholm H. Lindström M. Somervuo P. Heap J. Minton N. Lindén J.. (2011). *cspB* encodes a major cold shock protein in *Clostridium botulinum* ATCC 3502. Int. J. Food Microbiol. 146, 23–30. 10.1016/j.ijfoodmicro.2011.01.033 21367479

[B79] Suutari M. Laakso S. (1994). Microbial Fatty Acids and Thermal Adaptation. Crit. Rev. Microbiol. 20, 285–328. 10.3109/10408419409113560 7857519

[B80] Taboada B. Ciria R. Martinez-Guerrero C. E. Merino E. (2012). ProOpDB: prokaryotic operon database. Nucleic Acids Res. 40, D627–D631. 10.1093/nar/gkr1020 22096236 PMC3245079

[B81] Wattam A. R. Abraham D. Dalay O. Disz T. L. Driscoll T. Gabbard J. L.. (2014). PATRIC, the bacterial bioinformatics database and analysis resource. Nucleic Acids Res. 42, D581–D591. 10.1093/nar/gkt1099 24225323 PMC3965095

[B82] Wouters J. A. Kamphuis H. H. Hugenholtz J. Kuipers O. P. De Vos W. M. Abee T. (2000). Changes in glycolytic activity of *Lactococcus lactis* induced by low temperature. Appl. Environ. Microbiol. 66, 3686–3691. 10.1128/AEM.66.9.3686-3691.2000 10966377 PMC92207

[B83] Xia B. Ke H. Inouye M. (2001). Acquirement of cold sensitivity by quadruple deletion of the *cspA* family and its suppression by PNPase S1 domain in *Escherichia coli*. Mol. Microbiol. 40, 179–188. 10.1046/j.1365-2958.2001.02372.x 11298285

[B84] Xia B. Ke H. Shinde U. Inouye M. (2003). The role of RbfA in 16S rRNA processing and cell growth at low temperature in *Escherichia coli*. J. Mol. Biol. 332, 575–584. 10.1016/S0022-2836(03)00953-7 12963368

[B85] Yamanaka K. Zheng W. Crooke E. Wang Y.-H. Inouye M. (2001). CspD, a novel DNA replication inhibitor induced during the stationary phase in *Escherichia coli*. Mol. Microbiol. 39, 1572–1584. 10.1046/j.1365-2958.2001.02345.x 11260474

[B86] Ye J. Coulouris G. Zaretskaya I. Cutcutache I. Rozen S. Madden T. L. (2012). Primer-BLAST: a tool to design target-specific primers for polymerase chain reaction. BMC Bioinform. 13:134. 10.1186/1471-2105-13-134 22708584 PMC3412702

[B87] Yu G. Wang L.-G. Han Y. He Q.-Y. (2012). Clusterprofiler: an R package for comparing biological themes among gene clusters. J. Integr. Biol. 16, 284–287. 10.1089/omi.2011.0118 22455463 PMC3339379

