# Supplementary material for: Changes in Transcriptome of Yersinia pseudotuberculosis IP32953 Grown at 3 and 28°C Detected by RNA Sequencing Shed Light on Cold Adaptation
Source: Front Cell Infect Microbiol. 2018 Nov 27;8:416. doi: 10.3389/fcimb.2018.00416 (PMC6277586; doi:10.3389/fcimb.2018.00416)
Supplement: Supplementary file 2 [file Data_Sheet_2.docx]

Supplementary Material

Changes in Transcriptome of *Yersinia pseudotuberculosis* IP32953 Grown at 3°C and 28°C Detected by RNA Sequencing Shed Light on Cold Adaptation

Jussa-Pekka Virtanen, Riikka Keto-Timonen*, Kaisa Jaakkola, Noora Salin, Hannu Korkeala

*** Correspondence:**DVM, PhD Riikka Keto-Timonen
[riikka.keto-timonen@helsinki.fi](mailto:riikka.keto-timonen@helsinki.fi)

# Supplementary Figures

Supplementary Figure 1. Gene expression profile clustering of *Yersinia pseudotuberculosis* strain IP32953 flagellar genes that showed significantly more transcripts (logFC ≥ 2, FDR ≤ 0.05) at 3°C than 28°C at least once during growth. Flagellar were highly expressed at 3°C mainly at the beginning of growth and start of logarithmic phase. The darker the color in the heatmap, the more transcripts the gene showed at that growth point.

**Expression profiles of flagellar genes at 3°C**

Supplementary Figure 2. Gene expression profile clustering of *Yersinia pseudotuberculosis* strain IP32953 chemotaxis genes that showed significantly more transcripts (logFC ≥ 2, FDR ≤ 0.05) at 3°C than 28°C at least once during growth. Chemotaxis genes were highly expressed at 3°C mainly at the start of logarithmic phase. The darker the color in the heatmap, the more transcripts the gene showed at that growth point.

**Expression profiles of chemotaxis genes at 3°C**

**Expression profiles of tRNA genes at 3°C**

Supplementary Figure 3. Gene expression profile clustering of *Yersinia pseudotuberculosis* strain IP32953 tRNA genes that showed significantly more transcripts (logFC ≥ 2, FDR ≤ 0.05) at 3°C than 28°C at least once during growth. The darker the color in the heatmap, the more transcripts the gene showed at that growth point.
